# Metabolic Remodeling during Biofilm Development of Bacillus subtilis

**DOI:** 10.1128/mBio.00623-19

**Published:** 2019-05-21

**Authors:** Tippapha Pisithkul, Jeremy W. Schroeder, Edna A. Trujillo, Ponlkrit Yeesin, David M. Stevenson, Tai Chaiamarit, Joshua J. Coon, Jue D. Wang, Daniel Amador-Noguez

**Affiliations:** a Graduate Program in Cellular and Molecular Biology, University of Wisconsin—Madison, Madison, Wisconsin, USA; b Department of Bacteriology, University of Wisconsin—Madison, Madison, Wisconsin, USA; c Morgridge Institute for Research, Madison, Wisconsin, USA; d Department of Chemistry, University of Wisconsin—Madison, Madison, Wisconsin, USA; e Department of Biomolecular Chemistry, University of Wisconsin—Madison, Madison, Wisconsin, USA; f College of Agricultural & Life Sciences, University of Wisconsin—Madison, Madison, Wisconsin, USA; Institut Pasteur; University of Washington

**Keywords:** *Bacillus subtilis*, acetoin, biofilms, metabolism, metabolomics, proteomics, transcriptomics

## Abstract

Biofilms are structured communities of tightly associated cells that constitute the predominant state of bacterial growth in natural and human-made environments. Although the core genetic circuitry that controls biofilm formation in model bacteria such as Bacillus subtilis has been well characterized, little is known about the role that metabolism plays in this complex developmental process. Here, we performed a time-resolved analysis of the metabolic changes associated with pellicle biofilm formation and development in B. subtilis by combining metabolomic, transcriptomic, and proteomic analyses. We report surprisingly widespread and dynamic remodeling of metabolism affecting central carbon metabolism, primary biosynthetic pathways, fermentation pathways, and secondary metabolism. Most of these metabolic alterations were hitherto unrecognized as biofilm associated. For example, we observed increased activity of the tricarboxylic acid (TCA) cycle during early biofilm growth, a shift from fatty acid biosynthesis to fatty acid degradation, reorganization of iron metabolism and transport, and a switch from acetate to acetoin fermentation. Close agreement between metabolomic, transcriptomic, and proteomic measurements indicated that remodeling of metabolism during biofilm development was largely controlled at the transcriptional level. Our results also provide insights into the transcription factors and regulatory networks involved in this complex metabolic remodeling. Following upon these results, we demonstrated that acetoin production via acetolactate synthase is essential for robust biofilm growth and has the dual role of conserving redox balance and maintaining extracellular pH. This report represents a comprehensive systems-level investigation of the metabolic remodeling occurring during B. subtilis biofilm development that will serve as a useful road map for future studies on biofilm physiology.

## Abstract (precis)

Bacterial biofilms are ubiquitous in natural environments and play an important role in many clinical, industrial, and ecological settings. Although much is known about the transcriptional regulatory networks that control biofilm formation in model bacteria such as Bacillus subtilis, very little is known about the role of metabolism in this complex developmental process. To address this important knowledge gap, we performed a time-resolved analysis of the metabolic changes associated with bacterial biofilm development in B. subtilis by combining metabolomic, transcriptomic, and proteomic analyses. Here, we report a widespread and dynamic remodeling of metabolism affecting central carbon metabolism, primary biosynthetic pathways, fermentation pathways, and secondary metabolism. This report serves as a unique hypothesis-generating resource for future studies on bacterial biofilm physiology. Outside the biofilm research area, this work should also prove relevant to any investigators interested in microbial physiology and metabolism.

## INTRODUCTION

Biofilms are assemblages of tightly associated bacteria encapsulated by a self-produced extracellular matrix that allows attachment to surface and confers protection against environmental stressors (1–3). Bacterial biofilms are ubiquitous in natural and human-made environments, and their importance in clinical, industrial, ecological, and agricultural settings is widely recognized (4–10). Biofilms colonize biotic and abiotic surfaces in aquatic and soil environments (11–13). They also account for a large fraction of microbial infections in humans, including hospital-acquired infections (14–19).

The Gram-positive bacterium Bacillus subtilis is an established model system for investigating the molecular mechanisms of biofilm formation and development (1, 20–22). B. subtilis forms biofilms on plant roots (23, 24) and is utilized as a plant growth-promoting factor and a protective agent against plant bacterial pathogens (25, 26). B. subtilis is also found in the human gastrointestinal tract and has been proposed as a potential probiotic (27–29). Previous research provided insights into the core transcriptional regulatory network that controls biofilm development in this bacterium (1, 3); at the center of this regulatory network sits the master regulator Spo0A, whose phospho-activation by sensory kinases and phosphorelay proteins initiates a transcriptional cascade that results in inactivation of the biofilm repressors AbrB and SinR, upregulation of extracellular matrix (ECM) biosynthetic genes, and inhibition of flagellum-dependent motility and autolysin genes (30). In addition to Spo0A, the two-component system DegS-DegU influences biofilm formation by activating ECM and cell wall synthesis genes, metabolic genes, and multiple other targets of unknown function (31–35).

Despite our current understanding of the core biofilm transcriptional network, little is known about the metabolic changes that accompany this complex developmental process or their roles in driving biofilm formation. In addition, even though biofilms represent a predominant way of life for most bacteria, our current knowledge on bacterial metabolism and its regulation is derived almost exclusively from studies of exponentially growing planktonic cells, using domesticated strains deficient at forming biofilms. In recent years, these knowledge gaps have attracted increasing attention, and studies have begun to unravel the metabolism of bacterial biofilms. Some of the research on biofilm-associated metabolism has been focused on competition and cooperation in nutrient utilization (36–39). For example, a recent study described a metabolic codependence in B. subtilis colony biofilms in which growth in the biofilm periphery halts periodically to allow sufficient time for nutrient diffusion (i.e., diffusion of glutamate) to the sheltered interior cells, which then release ammonium that diffuses to outer cells (38). Also, a study in Shewanella oneidensis recently showed increased activity of one-carbon metabolism in biofilms versus planktonic cells (40). An active research area related to biofilm metabolism has been the identification of metabolites that trigger or inhibit biofilm formation. Studies have shown that plant polysaccharides (24), malic acid (41), and acetic acid (22), among other factors, promote B. subtilis biofilm formation whereas others such as d-amino acids (42) and cyclic-di-AMP (43) inhibit it. The mechanisms by which these molecules influence B. subtilis biofilm are currently understudied (44–46).

Here, to advance our understanding of biofilm metabolism, we performed a time-resolved analysis of metabolic changes associated with pellicle biofilm formation and development in B. subtilis by combining metabolomic, transcriptomic, and proteomic analyses. We found surprisingly widespread and dynamic remodeling of metabolism affecting central carbon metabolism, primary biosynthetic pathways, fermentation pathways, and secondary metabolism. Early biofilm development was characterized by upregulation of energy-generating and biosynthetic pathways, followed by upregulation of catabolic processes in later development. A subset of metabolic alterations was associated with limited availability of specific nutrients, and others appeared related to microbial competition. Our results indicate that remodeling of metabolism during biofilm development was generally controlled at the transcriptional level, and we provide insights into the transcription factors (TFs) and regulatory networks involved. Finally, we provide examples of the relevance of metabolic remodeling during biofilm development by demonstrating that upregulation of acetoin production is essential for robust biofilm growth.

## RESULTS

### Biofilm growth and sample handling.

**FIG 1:**
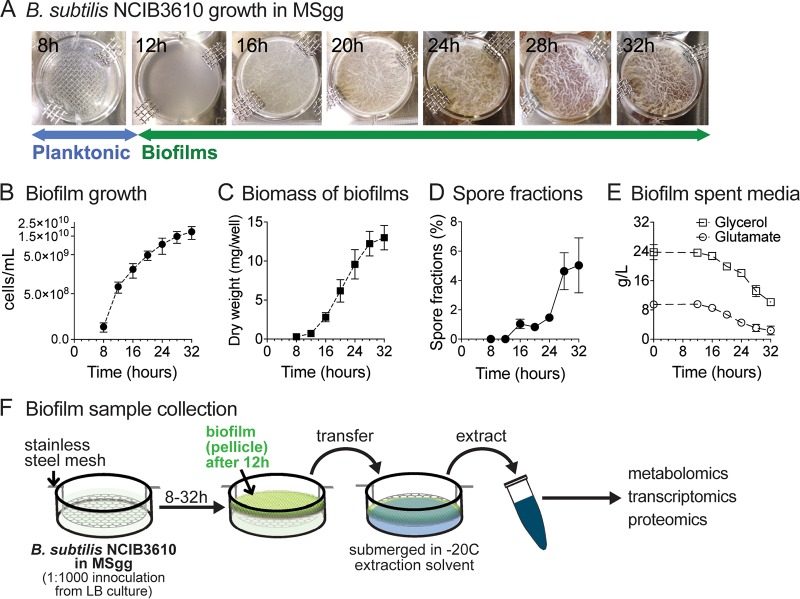
Biofilm growth and sample handling. (A) B. subtilis NCIB3610 was inoculated into modified MSgg media at time *t* = 0. Standing cultures at 8 h after inoculation were planktonic. Initial pellicle formation (i.e., fragile biofilms) reproducibly took place at approximately 12 h. By 16 h, pellicles became noticeably more robust and the wrinkled morphology started to become apparent. Biofilm development was monitored until 32 h of growth. The pictures shown are representative of all experiments performed. (B) Biofilm growth was quantified in cells per milliliter (direct cell counts). Data represent averages of results from 4 to 6 biological replicates ± standard errors of the means (SEM). (C) Biomass of B. subtilis biofilms. Extracted pellicles were air-dried and weighed. Data represent averages of results from 3 biological replicates ± SEM. (D) Spore fractions (percent) of biofilms. Data represent averages of results from 5 biological replicates ± SEM. (E) Glycerol and glutamate concentrations in spent biofilm media over the course of biofilm growth. Data represent averages of results from 3 biological replicates ± SEM. (F) Sample collection for metabolomic, transcriptomic, and proteomic analyses was performed at 4-h intervals from 8 to 32 h of growth at 37°C. Planktonic cells (8 h) were collected via rapid filtration. Biofilm samples (12 to 32 h) were collected by lifting a custom-made stainless-steel mesh and transferring the pellicle to the appropriate extraction solvent or buffer.

Bacillus subtilis strain NCIB3610 (here referred to as “B. subtilis”) forms robust biofilms under a variety of growth conditions in natural environments and laboratory settings, including on solid growth substrates (e.g., agar plates) and at the air-liquid interface of standing liquid cultures (20). Air-liquid-interface biofilms, commonly referred to as pellicles, constitute a well-established experimental system for investigating biofilm formation and development in B. subtilis; as a standing culture reaches a certain cell density (approximately 3 × 10^7^ CFU/ml), planktonic cells migrate toward the air-liquid interface, forming an intricate, floating pellicle structure (i.e., a biofilm) (20, 47). Using B. subtilis pellicle formation, we sought to characterize metabolic alterations associated with biofilm formation and development and underlying transcriptional regulation by combining metabolomics (liquid chromatography-mass spectrometry [LC-MS]), transcriptome sequencing (RNA-sequencing), and proteomics (LC-tandem MS [LC-MS/MS]) analyses. Our experimental system consisted of standing cultures (12-well plate format) grown at 37°C in a defined medium containing glycerol and glutamate as the sole carbon and nitrogen sources (20). As depicted in Fig. 1A, turbidity (optical density at 600 nm [OD_600_] of approximately 0.06 to 0.08) of planktonic cultures was observed approximately 8 hours (h) after initial culture inoculation (*t* = 0 h). Initial pellicle formation (i.e., formation of fragile biofilms) reproducibly took place at approximately 12 h. By 16 h, pellicles became noticeably more robust, and the wrinkled morphology started to become apparent. The wrinkled and folded architectures became more pronounced as the biofilms matured at between 20 and 32 h. Cell numbers increased steadily over the time course and reached roughly 2* × *10^10^ cells/ml by 32 h of growth (Fig. 1B); pellicle biomass accumulation closely followed growth (Fig. 1C); spore fractions remained low (<2%) through most of development but increased to ∼5% at 28 to 32 h (Fig. 1D). Samples for intracellular and extracellular metabolomic analyses, gene expression analysis, and shotgun proteomics analyses were collected at 8 to 32 h at 4-h intervals (7 time points) (Fig. 1F).

### Metabolome analysis reveals global and dynamic changes in intracellular metabolite levels during biofilm development.

**FIG 2:**
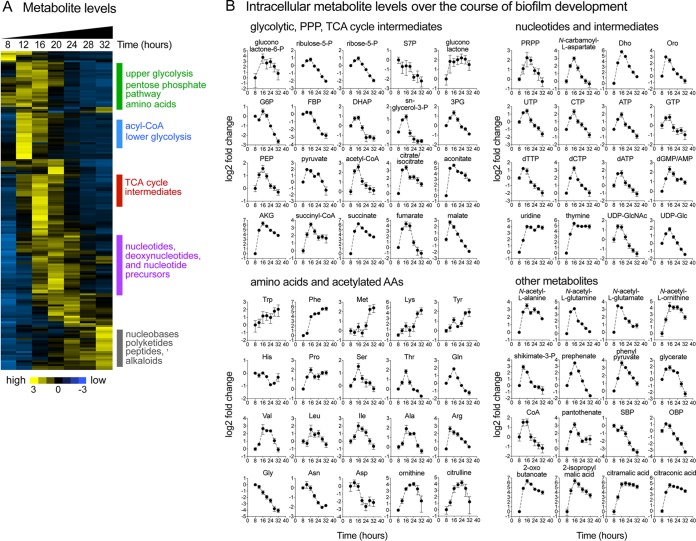
Dynamic remodeling of B. subtilis metabolome during biofilm development. (A) Samples for LC-MS metabolomic analysis were taken at 8, 12, 16, 20, 24, 28, and 32 h during biofilm development. Metabolite measurements were normalized by cell numbers and are displayed as log2 z-scores. The heat map displays data for 166 metabolites. The yellow (high) and blue (low) color scale indicates relative metabolite abundances across the time course. (B) Intracellular levels of selected metabolites displayed as log_2_ fold changes relative to the initial 8-h time point. Data represent averages of results from 4 biological replicates ± SEM. Abbreviations: P, phosphate; S7P, sedoheptulose-7-phosphate; G6P, glucose-6-phosphate; FBP, fructose-1,6-bisphosphate; DHAP, dihydroxy-acetone-phosphate; 3PG, 3-phosphoglycerate; PEP, phosphoenolpyruvate; AKG, α-ketoglutarate; Trp, tryptophan; Phe, phenylalanine; Met, methionine; Lys, lysine; Tyr, tyrosine; His, histidine; Pro, proline; Ser, serine; Thr, threonine; Gln, glutamine; Val, valine; Leu, leucine; Ile, isoleucine; Ala, alanine; Arg, arginine; Asn, asparagine; Gly, glycine; Asp, aspartate; PRPP, 5-phosphoribosyl-pyrophosphate; Dho, dihydroorotate; Oro, orotate; GlcNAc, *N*-acetylglucosamine; Glc, glucose; SBP, sedoheptulose bisphosphate; OBP, octulose bisphosphate.

Intracellular metabolomic analyses of biofilm samples were performed using a set of LC-MS methods (48–51) that together enable measurement of over 200 metabolites. Of the analyzed metabolites, 166 were reproducibly measured, and 127 displayed significant alterations in their intracellular levels over the course of biofilm formation and development (*P* < 0.05; analysis of variance [ANOVA]) (see Table S1A at https://bit.ly/2XrPH9Z). Our analysis revealed remarkably dynamic changes during biofilm development in most of the measured metabolic pathways (Fig. 2A). These alterations encompassed both primary metabolic pathways such as glycolysis, pentose phosphate pathway (PPP), tricarboxylic acid (TCA) cycle, and nucleotide and amino acid biosynthesis pathways and secondary metabolic pathways such as acetoin, pulcherrimin, and bacillibactin biosynthesis pathways, among others.

Various interesting trends were evident in the profiles of metabolites belonging to the same class or the same pathway (Fig. 2B). For example, in primary metabolism, the levels of TCA cycle intermediates (i.e., citrate, aconitate, α-ketoglutarate [α-KG], succinyl-coenzyme A [succinyl-CoA], succinate, malate, and fumarate) displayed a sharp increase during early biofilm development (12 to 16 h). Levels of branched-chain amino acids (leucine/isoleucine and valine) peaked during middle biofilm development (∼20 h) whereas those of aromatic amino acids (phenylalanine, tyrosine, and tryptophan) increased steadily over time. Levels of upper glycolytic intermediates (e.g., glucose 6-phosphate, fructose1,6-bisphosphate, and dihydroxyacetone phosphate) and PPP intermediates (ribose 5-phosphate, ribulose 5-phosphate, and sedoheptulose 7-phosphate) displayed continued decreases starting at 20 to 24 h. Nucleotide triphosphates (ATP, GTP, UTP, and CTP) and nucleotide biosynthetic precursors (e.g., 5-phosphoribosyl-pyrophosphate [PRPP], *N*-carbamoyl-l-aspartate, orotate, and dihydroorotate) displayed maximum levels at around 16 to 24 h, while the levels of nucleotide salvage intermediates (e.g., nucleosides and nucleobases) increased in late biofilm development. In secondary metabolism, levels of *N*-acetylated amino acids (e.g., *N*-acetyl-glutamine and *N*-acetyl-glutamate) and nucleotide sugars (e.g., UDP-glucose [Glc], UDP-GlcNAc, and ADP-Glc) displayed transient increases during early biofilm development (∼12 h).

We also examined secreted metabolites (see Table S1B at https://bit.ly/2XrPH9Z). Interestingly, our analysis revealed levels of extracellular accumulation of the TCA cycle intermediates fumarate and malate during early to middle biofilm development that roughly paralleled their intracellular levels. Several other metabolites accumulated extracellularly starting at ∼20 h. These included the siderophore bacillibactin, the iron-chelating molecule pulcherriminic acid and its precursor cyclo(l-leucyl-l-leucyl) (cLL), and several *de novo* pyrimidine biosynthetic intermediates (i.e., *N*-carbamoyl-l-aspartate, dihydroorotate, and orotate).

### Transcriptomic and proteomic analyses revealed widespread remodeling of metabolism during biofilm development.

In parallel to metabolomic measurements, we performed genome-wide analyses of changes in gene expression (RNA-sequencing) and protein abundance (shotgun proteomics) during B. subtilis biofilm development. We measured the transcript abundances of 4,348 genes, including 4,246 on the chromosome and 102 on the pBS32 macroplasmid, representing coverage of 97.6% of the B. subtilis genome (52). Remarkably, 2,477 genes (∼57% of the genome; 47 genes were plasmid borne) showed significant alterations in their transcript levels during biofilm development (*P* < 0.001; ANOVA; false-discovery rate [FDR] = 0.00175) (see Table S2A at https://bit.ly/2Dn2HX2).

For protein quantification, we employed high-resolution tandem mass spectrometry (MS/MS) coupled to high-pressure liquid chromatography. We collected a total of 4,422,846 MS/MS spectra, of which 1,478,117 were mapped to peptide sequences (i.e., PSMs). The throughput and depth from these shotgun proteomics analysis were driven by recent improvements in sample preparation, MS technology, and peptide separation by high pressure chromatography (53–55, 136, 137, 144). Altogether, we identified over 28,000 unique peptide sequences, which mapped to 2,582 proteins (2,474 with measured transcript levels). Of the measured proteins, 875 displayed significant changes in abundance during biofilm development (*P* < 0.001; ANOVA; FDR = 0.00295). Of these, 545 had correspondingly significant changes in transcript levels with *P* values of <0.001, while the rest had *P* values between 0.01 and 0.001 (see Table S3 at https://bit.ly/2XqQAzF).

**FIG 3:**
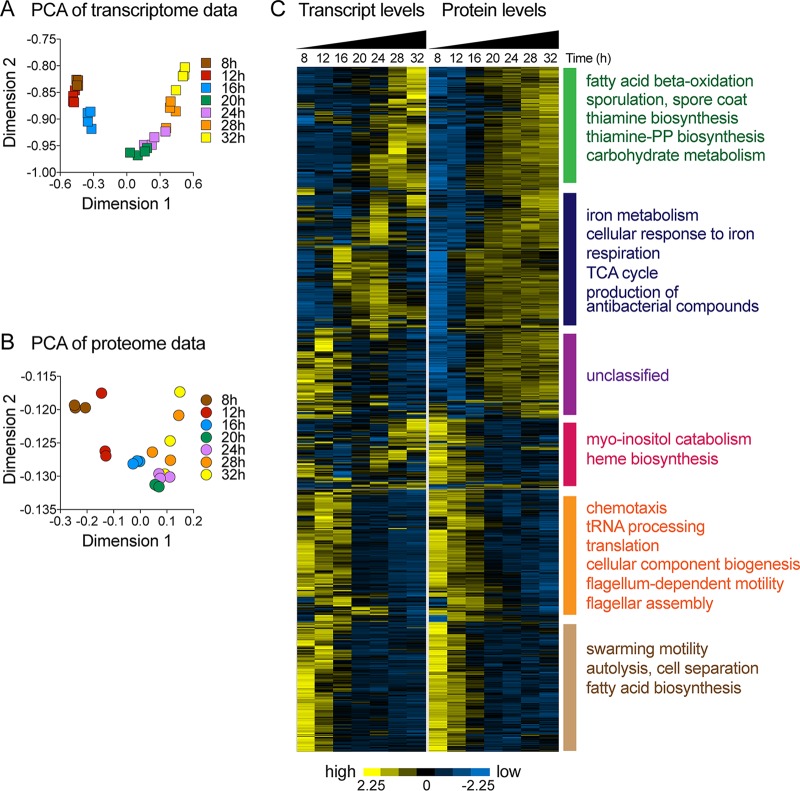
Transcriptome and proteome of B. subtilis biofilms. Samples for transcriptomic and proteomic analyses were taken, independently, at 8, 12, 16, 20, 24, 28, and 32 h during biofilm development. (A and B) Principal-component analysis (PCA) of transcriptomics (A) and proteomics (B) data. (C) Relative transcript and protein levels of genes with significant changes in protein abundance (*P* value, <0.001; FDR = 0.00295; ANOVA) are displayed. Each row across both heat maps represents a single gene. Transcript and protein levels are displayed as log_2_ z-scores. The yellow (high) and blue (low) color scale indicates relative transcript or protein levels across the time course. Enrichment of functional categories was determined using gene ontology analysis (143).

Panels A and B of Fig. 3 show results of principal-component analyses (PCA) of transcriptomics and proteomics data. Biological replicates mostly clustered together, and samples from different time points were well ordered and gradually distributed across the first two major components, indicating progressive changes in gene expression and protein abundance along the time course of biofilm development.

Panel C of Fig. 3 displays results from an unsupervised hierarchical clustering analysis of the 875 proteins with significant changes in abundance during biofilm development alongside their corresponding transcript levels. We observed excellent agreement between changes in protein abundance and transcript levels for the majority of genes. In several instances, a temporary increase in the transcript level resulted in a sustained increase in protein abundance. A wide range of cellular processes were affected by changes in transcript and protein levels, many of them related to metabolism. For example, fatty acid synthesis, terpenoid biosynthesis, flagellar biogenesis, chemotaxis, and autolysis were downregulated during biofilm development. Genes involved in cell wall synthesis, sporulation, extracellular matrix production, carbohydrate metabolism, acetoin metabolism, fatty acid beta-oxidation, thiamine biosynthesis, iron metabolism, and antibiotic compound synthesis were upregulated. Glycolysis, TCA cycle, nucleotide synthesis, and amino acid synthesis genes displayed more-complex expression trajectories.

### Metabolic remodeling during B. subtilis biofilm development.

Our integrated metabolomic-transcriptomic-proteomic analysis revealed a widespread and dynamic remodeling of metabolism during B. subtilis biofilm development that affected central carbon metabolism, primary biosynthetic pathways, fermentation pathways, and secondary metabolism. We summarize a subset of the most significant alterations below.

### Upregulation of the TCA cycle during early biofilm development.

**FIG 4:**
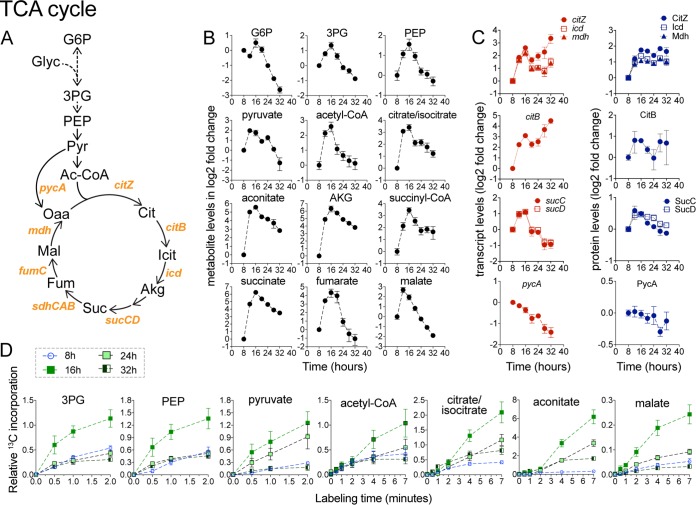
Upregulation of the TCA cycle in early biofilm development. (A) TCA cycle pathway and enzymes. Abbreviations: Glyc, glycerol; PEP, phosphoenoloyruvate; Pyr, pyruvate; Ac-CoA, acetyl-CoA; Icit, isocitrate; Akg, α-ketoglutarate; Suc, succinate; Fum, fumarate; Mal, malate; Oaa, oxaloacetate. (B) Intracellular levels of TCA cycle intermediates displayed as log_2_ fold changes relative to the initial 8-h time point. Data represent averages of results from 4 biological replicates ± SEM. (C) Levels of TCA cycle transcripts and proteins encoded by the *citZ-icd-mdh*, *citB*, and *sucCD* operons. Also shown is *pycA*, encoding pyruvate carboxylase. Genes within the same operon are shown in the same graph. Data are displayed as log2 fold changes relative to the initial 8-h time point and represent averages of results from 4 (transcript) or 3 (protein) biological replicates ± SEM. (D) Relative levels of ^13^C-carbon incorporation from ^13^C-glycerol into TCA and lower glycolytic intermediates at 8, 16, 24, and 32 h during biofilm development. Data represent averages of results from 3 biological replicates ± SEM.

A unifying theme of the metabolic remodeling during early biofilm development was an upregulation of energy-generating and biosynthetic pathways. Intracellular levels of TCA cycle intermediates displayed a rapid and highly coordinated increase in early biofilm development, peaking around 12 to 16 h before decreasing at various rates at subsequent time points (Fig. 4B). These alterations in metabolite levels correlated with the increased abundance of TCA enzymes (Fig. 4C). Specifically, the results seen with the *citZ-icd-mdh* operon (encoding citrate synthase, isocitrate dehydrogenase, and malate dehydrogenase) displayed an increase in transcript and protein abundance that matched the early increase in TCA cycle intermediates. Similarly, transcript and protein levels of succinyl-CoA synthetase (*sucCD* operon) increased in early biofilm development. Aconitase (*citB*) transcript levels also increased early on, although the abundance of the protein increased only modestly.

To further investigate changes in TCA cycle activity, we performed dynamic isotope tracer experiments at 8, 16, 24, and 32 h using ^13^C-glycerol. In agreement with increased levels of TCA cycle intermediates and increased enzyme abundance, dynamic labeling experiments indicated that carbon flux into the TCA cycle, via both citrate synthase and anaplerotic reactions, increased considerably at 16 h before decreasing somewhat at 24 and 32 h of growth (Fig. 4D; see also Fig. S1 in the supplemental material). Increased carbon flux into the TCA cycle was matched by increased flux into lower glycolytic intermediates (Fig. 4D). Taken together, our data show that TCA cycle activity is rapidly upregulated during early biofilm development (12 to 16 h), which may be critical for providing energy (i.e., ATP and GTP), reducing power [i.e., NAD(P)H] and precursors to biosynthetic pathways and cellular processes involved in early biofilm development.

10.1128/mBio.00623-19.1 FIG S1 ^13^C incorporation from ^13^C-labeled glycerol into malate. ^13^C-carbon incorporation from ^13^C-glycerol into malate at 8, 16, 24, and 32 h during biofilm development was analyzed. Line graphs show primary forms of ^13^C-labeled malate. Data represent averages of results from 3 biological replicates ± SEM. Download FIG S1, TIF file, 16.7 MB. Copyright © 2019 Pisithkul et al. 2019 Pisithkul et al. This content is distributed under the terms of the Creative Commons Attribution 4.0 International license.

### Alterations in *de novo* nucleotide biosynthesis.

**FIG 5:**
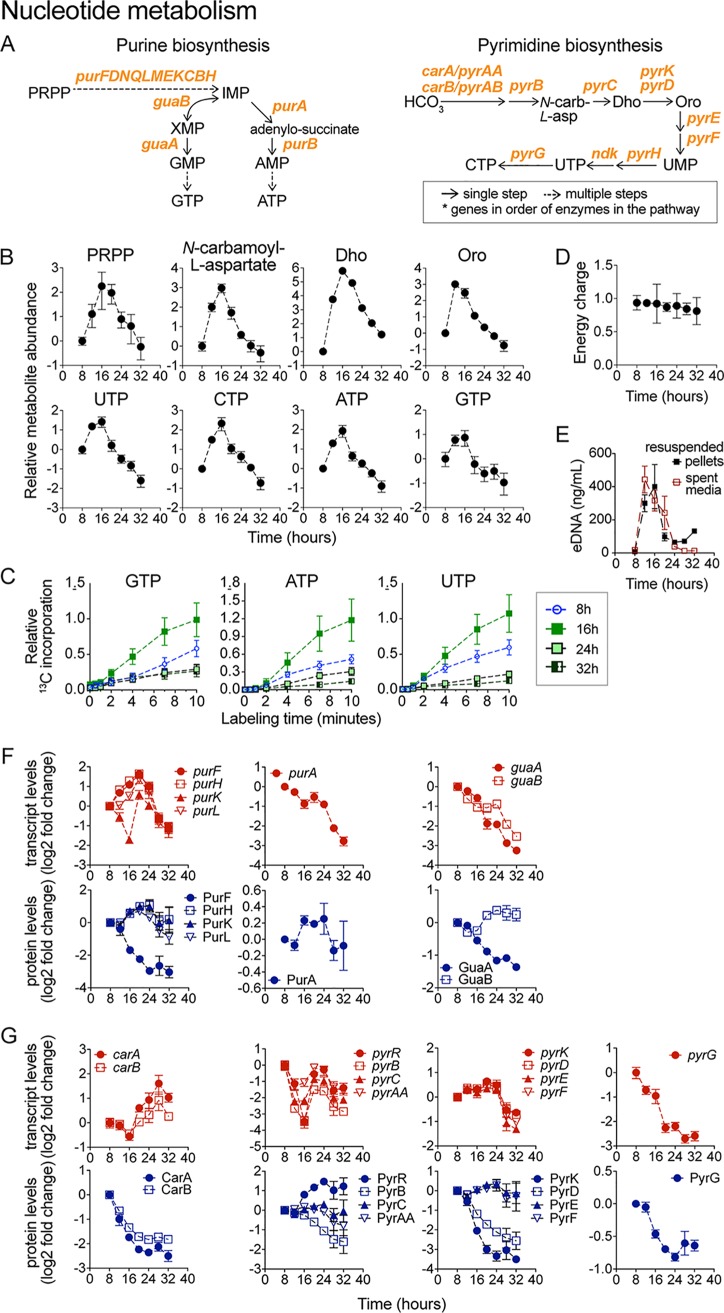
Nucleotide metabolism during biofilm development. (A) B. subtilis purine and pyrimidine biosynthetic pathways. Abbreviations: HCO_3_, biocarbonate; *N*-carb-l-asp., *N*-carbamoyl-l-aspartate; Dho, dihydroorotate; Oro, orotate; PRPP, 5-phosphoribosyl-pyrophosphate; IMP, inosine-monophosphate; XMP, xanthosine-monophosphate. (B) Intracellular levels of GTP, ATP, and UTP displayed as log_2_ fold changes relative to the initial 8-h time point. Data represent averages of results from 4 biological replicates ± SEM. (C) Relative levels of ^13^C-carbon incorporation into GTP, ATP, and UTP at 8, 16, 24, and 32 h during biofilm development. Data represent averages of results from 3 biological replicates ± SEM. (D) Energy charge. (E) Extracellular DNA (eDNA) in spent media of biofilms and resuspended biofilm pellets. (F and G) Transcript and protein levels of purine (F) and pyrimidine (G) biosynthetic genes. Genes within the same operon are shown in the same graph. Data are displayed as log_2_ fold changes relative to the initial 8-h time point. Data represent averages of results from 4 (transcript) or 3 (protein) biological replicates ± SEM.

Upregulation of the TCA cycle was concurrent with increased levels of nucleotides, deoxynucleotides, and their biosynthetic intermediates. The intracellular levels of nucleotides (NTPs) and of their biosynthetic precursors followed highly similar profiles during biofilm development (Fig. 5B). They all increased early in biofilm development, reached a peak at 16 h, and declined gradually afterward. This coordinated pattern indicated upregulation of nucleotide biosynthesis during early biofilm growth. In agreement with this, dynamic ^13^C-glycerol tracer experiments revealed an increased rate of ^13^C-carbon incorporation into purines and pyrimidines at 16 h (Fig. 5C). The adenylate energy charge remained nearly constant and close to optimal levels at all times despite these large fluctuations in nucleotide levels, which indicated adequate energy availability throughout biofilm development (Fig. 5D). Interestingly, the concentration of extracellular DNA (eDNA), a key component of the ECM (56, 57), followed a trend that was nearly identical to that seen with the intracellular nucleotides and biosynthetic precursors (Fig. 5E), suggesting that upregulation of nucleotide biosynthesis supports eDNA production.

Matching the increase in purine nucleotide levels, the abundance of the enzymes initially involved in purine biosynthesis (i.e., *pur* operon and IMP production) increased in early biofilm development (Fig. 5F). However, the abundance of subsequent enzymes in the pathway required for the conversion of IMP to ATP or GTP (e.g., Gmk, GuaA, and Adk) decreased steadily throughout biofilm development (Fig. S2). Therefore, the concerted early increases in purine nucleotide levels and in the levels of the corresponding biosynthetic precursors can be explained only partially by the increases in the enzyme levels.

10.1128/mBio.00623-19.2 FIG S2 Expression of nucleotide metabolism genes during biofilm development. (A) Purine biosynthesis. (B) Pyrimidine biosynthesis. (C) Transcript and protein levels of purine biosynthesis genes displayed as log_2_ fold changes relative to the initial 8-h time point. Error bars represent ± SEM of results from 4 (transcript) and 3 (protein) biological replicates. (D) Transcript and protein levels of pyrimidine biosynthesis genes displayed as log_2_ fold changes relative to the initial 8-h time point. Error bars represent ± SEM of results from 4 (transcript) and 3 (protein) biological replicates. Download FIG S2, TIF file, 16.3 MB. Copyright © 2019 Pisithkul et al. 2019 Pisithkul et al. This content is distributed under the terms of the Creative Commons Attribution 4.0 International license.

The levels of most enzymes in the pyrimidine biosynthetic pathway declined during biofilm development (Fig. 5G; see also Fig. S2). For example, protein levels of CarA, CarB, PyrB, and PyrD, required for UMP production, displayed significant decreases over time. Similarly, protein levels of PyrH and PyrG, required to produce UTP and CTP from UMP, decreased rapidly during early biofilm development. The disparity between the increased levels of pyrimidine nucleotides and biosynthetic precursors and the enzyme levels during early biofilm development suggests a dominant role for metabolic regulation (e.g., substrate availability) or posttranslational regulation (e.g., allostery) at that stage. Despite these early complexities, the long-term trend for both purine biosynthesis and pyrimidine biosynthesis was that of downregulation over time, which matches the generalized decrease in nucleotides and deoxy-nucleotide levels and the decreased rate of ^13^C-carbon incorporation that we observed in late biofilm development (Fig. 5C).

### Transient upregulation of extracellular matrix (ECM) synthesis.

**FIG 6:**
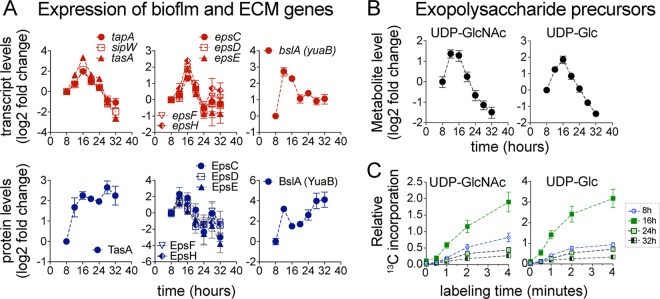
Upregulation of extracellular matrix (ECM) genes. (A) Transcript and protein levels of ECM genes displayed as log_2_ fold changes relative to the initial 8-h time point. Error bars represent ± SEM of results from 4 (transcript) and 3 (protein) biological replicates. (B) Intracellular levels of UDP-GlcNAc and UDP-Glc, precursors of exopolysaccharide biosynthesis, displayed as log_2_ fold changes relative to the initial 8-h time point. Data represent averages of results from 4 biological replicates ± SEM. (C) Relative levels of ^13^C-carbon incorporation into UDP-GlcNAc and UDP-Glc at 8, 16, 24, and 32 h during biofilm development. Data represent averages of results from 3 biological replicates ± SEM.

The upregulation of extracellular matrix (ECM) genes (*epsA-O* [genes from *epsA* to *epsO*], *tapA-sipW-tasA*, and *bslA* operons) and the increase in the levels of ECM biosynthetic intermediates UDP-Glc and UDP-GlcNAc were also concurrent with the upregulation of the TCA cycle (Fig. 4; see also Fig. 6). Interestingly, the *epsA-O* and *tapA-sipW-tasA* operons displayed only a transient increase in expression (peaking at 16 h) rather than the sustained increase that might be expected for ECM genes in growing biofilms (Fig. 6A). The transient upregulation of the *epsA-O* operon matched the transient increase in UDP-Glc and UDP-GlcNAc levels (Fig. 6B) and the transient increase in ^13^C-carbon incorporation into these metabolites at 16 h (Fig. 6C). Despite the transient transcriptional upregulation of *tapA-sipW-tasA*, we observed a sustained increase in protein levels of TasA (Fig. 6A), the major proteinaceous component of ECM (58), that persisted throughout biofilm development. This observation indicated that TasA production, after its initial induction at 12 h, continued throughout the 32-h time course and was maintained at a fairly constant proportion of the total protein between 16 to 32 h. This is consistent with our observation that the total protein in the pellicles and spent media increased continuously and roughly proportionally with biofilm growth (Fig. S3A). It also agreed with the impaired biofilm growth and decreased extracellular protein content displayed by the *ΔtasA* mutant (Fig. S3B, C, and D). Another of the major proteinaceous components of the ECM, BslA, which forms a water-repellent surface layer in the biofilm (59), was also continuously produced during biofilm growth (Fig. 6A).

10.1128/mBio.00623-19.3 FIG S3 Biofilm protein content, extracellular iron concentration, and expression of motility and chemotaxis genes. (A) Protein content in pellicle biofilms and spent medium of B. subtilis NCIB 3610. Data represent averages of results from 3 and 4 biological replicates ± SEM for pellicles and spent medium samples, respectively. (B) Pellicle biofilm development of WT versus *ΔtasA* mutant biofilms. (C) Total biomass of WT versus *ΔtasA* mutant biofilms. Data represent averages of results from 3 to 6 biological replicates ± SEM. (D) Extracellular protein concentrations measured from spent media of WT versus *ΔtasA* mutant biofilms. Data represent averages of results from 4 and 3 biological replicates ± SEM for the WT and *ΔtasA* strains, respectively. (E) Total iron concentrations (in micromoles) in the spent medium of WT biofilms. Data represent averages of results from 3 biological replicates ± SEM. (F) Protein levels of motility and flagellar proteins displayed as log_2_ fold changes relative to the initial 8-h time point. Data represent averages of results from 3 biological replicates ± SEM. Download FIG S3, TIF file, 33.5 MB. Copyright © 2019 Pisithkul et al. 2019 Pisithkul et al. This content is distributed under the terms of the Creative Commons Attribution 4.0 International license.

### Amino acid metabolism during biofilm development.

Intracellular amino acid levels displayed complex and dynamic behaviors (Fig. 2B). Levels of serine, threonine, alanine, glutamine, branched-chain amino acids, and acetylated amino acids peaked around 16 to 20 h of growth. Levels of methionine, lysine, and aromatic amino acids increased during biofilm development. A few amino acids, such as glycine and asparagine, decreased steadily in abundance over time. Despite these diverse trends, ^13^C-tracer experiments indicated increased carbon incorporation into various amino acids and acetylated amino acids at 16 to 24 h in biofilm development (Fig. S4A). Transcript and protein profiles of amino acid biosynthesis genes were also complex but were, in several instances, reminiscent of those seen in purine biosynthesis: displaying a small increase in expression early in biofilm development before decreasing in expression at later time points (Fig. S4B). As shown in Fig. 1E, extracellular glutamate levels decreased gradually during biofilm growth. Transcript and protein levels of glutamate/glutamine metabolism genes and ammonia assimilation pathways displayed complex trends, but a compensatory response to decreased glutamate availability might be the upregulation of the *gltAB* operon, encoding glutamate synthase, and *gltC*, encoding a transcriptional activator via the *gltAB* operon (60) (Fig. S4B and C). The concerted upregulation of TCA cycle activity, nucleotide biosynthesis, and amino acid synthesis during early biofilm development represents a coordinated response that may support ECM production and other biofilm-specific processes during early development.

10.1128/mBio.00623-19.4 FIG S4 Amino acid metabolism. (A) ^13^C-carbon incorporation from ^13^C-glycerol into amino acids at 8, 16, 24, and 32 h during biofilm development. Data represent averages of results from 3 biological replicates ± SEM. (B) Transcript and protein levels of amino acid biosynthetic genes. Genes within the same operon are shown in the same graph. Data are displayed as log_2_ fold changes relative to the initial 8-h time point. Data represent averages of results from 4 (transcript) or 3 (protein) biological replicates ± SEM. (C) Glutamate/glutamate biosynthesis and ammonia assimilation pathways. Download FIG S4, TIF file, 11.4 MB. Copyright © 2019 Pisithkul et al. 2019 Pisithkul et al. This content is distributed under the terms of the Creative Commons Attribution 4.0 International license.

### Upregulation of iron acquisition and switch in electron-transfer proteins.

Some of the metabolic alterations that we observed during biofilm development appeared to address challenges associated with transport or availability of specific nutrients within different regions of the biofilm. Two prominent examples of this are the alterations related to iron assimilation and the upregulation of acetoin production (see below).

**FIG 7:**
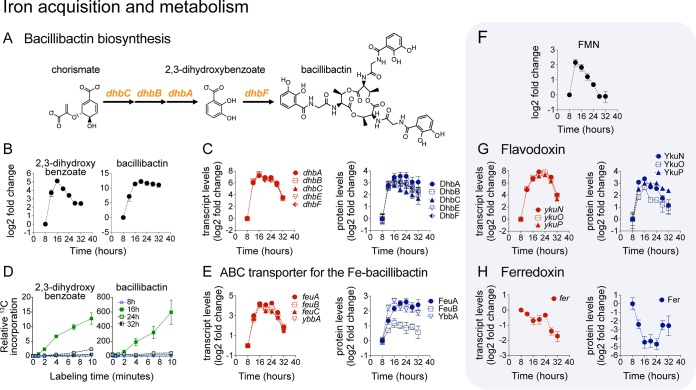
Upregulation of iron acquisition during biofilm development. (A) Bacillibactin synthesis pathway. (B) Intracellular levels of 2,3-dihydroxybenzoate and bacillibactin displayed as log_2_ fold changes relative to the initial 8-h time point. Data represent averages of results from 4 biological replicates ± SEM. (C and E) Transcript and protein levels of bacillibactin synthesis (C) and ABC transporter genes (E) displayed as log_2_ fold changes relative to the initial 8-h time point. Data represent averages of results from 4 (transcript) or 3 (protein) biological replicates ± SEM. (D) Relative levels of ^13^C-carbon incorporation into 2,3-dihydroxybenzoate and bacillibactin at 8, 16, 24, and 32 h during biofilm development. Data represent averages of results from 3 biological replicates ± SEM. (F) Intracellular levels of flavin mononucleotide (FMN) displayed as log_2_ fold change relative to the initial 8-h time point. Data represent averages of results from 4 biological replicates ± SEM. FMN acts as cofactor for flavodoxin. (G and H) Transcript and protein levels of flavodoxin (G) and ferredoxin (H) displayed as log_2_ fold changes relative to the initial 8-h time point. Data represent averages of results from 4 (transcript) or 3 (protein) biological replicates ± SEM.

**(i) Upregulation of bacillibactin biosynthesis and transport.** Bacillibactin binds extracellular iron and transports it across the cell membrane via ATP-binding cassette (ABC) transporters. Bacillibactin is comprised of three 2,3-dihydroxybenzoate-glycine-threonine subunits assembled by a nonribosomal peptide synthetase encoded by the *dhbACEBF* operon (61) (Fig. 7A). We observed striking upregulation (∼180-fold increase) of *dhbACEBF* transcript levels at the beginning of biofilm development (^12 to 16^ h). This increase in transcript levels correlated with a significant (∼12-fold) increase in protein abundance, as well as with production and secretion of bacillibactin and intracellular accumulation of its biosynthetic precursor 2,3-dihydroxybenzoate (Fig. 7B and C). A dramatic increase in the rate of ^13^C-carbon incorporation into 2,3-dihydroxybenzoate and bacillibactin was also observed at 16 h (Fig. 7D), indicating that the maximum rate of bacillibactin synthesis occurred early in biofilm development. Matching the upregulation of bacillibactin biosynthesis, we observed a significant increase in expression (transcript and protein) of the ferri-bacillibactin ABC transporter encoded by *feuABC* (62) (Fig. 7E). The concerted upregulation of the siderophore bacillibactin and its corresponding ABC transporters during biofilm development indicates a need for biofilm cells to improve iron assimilation.

**(ii) Switch in electron-transfer proteins from ferredoxin to flavodoxin.** Flavodoxins are soluble electron shuttle proteins known to replace ferredoxin (Fer) under conditions of iron limitation (63). The expression profile of the *ykuNOP* operon, encoding three different flavodoxins, displayed a drastic upregulation in transcript (∼250-fold increase) and protein (∼8-fold increase) levels (Fig. 7G). This pattern of expression was nearly identical to that of the *dhb* operon. Intracellular levels of the flavodoxin cofactor flavin mononucleotide (FMN) correlated with YkuNOP protein levels (Fig. 7F). In contrast, Fer levels decreased during biofilm development, following a profile nearly opposite that seen with the flavodoxin levels (Fig. 7H).

### Upregulation of pulcherrimin and antibiotic synthesis.

**FIG 8:**
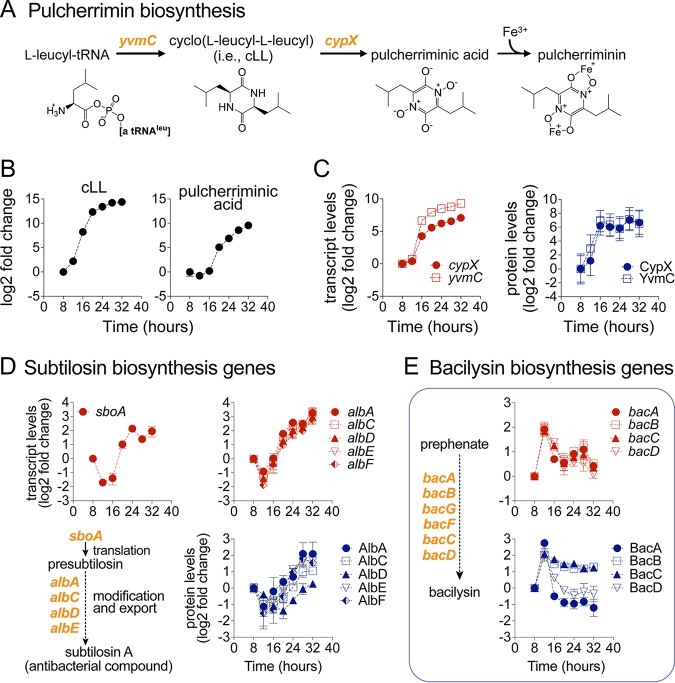
Upregulation of pulcherrimin and antibiotic biosynthesis. (A) Pulcherrimin biosynthesis pathway. (B) Extracellular levels of the biosynthetic precursor cyclo(l-leucyl-l-leucyl) (cLL) and pulcherriminic acid displayed as log_2_ fold changes relative to the initial 8-h time point. Data represent averages of results from 4 biological replicates ± SEM. (C) Transcript and protein levels of pulcherrimin synthesis (*yvmC-cypX* operon) displayed as log_2_ fold changes relative to the initial 8-h time point. Data represent averages of results from 4 (transcript) or 3 (protein) biological replicates ± SEM. (D and E) Transcript and protein levels of antibiotic synthesis genes. (D) *sboA-sboX-albABCDEFG* is a 9-gene operon encoding subtilosin-A biosynthetic enzymes and immunity against this compound. (E) *bacABCDEF* and *bacG* encode enzymes catalyzing biosynthesis of the antibiotic compound bacilysin. Data are displayed as log_2_ fold changes relative to the initial 8-h time point. Data represent averages of results from 4 (transcript) or 3 (protein) biological replicates ± SEM.

In natural environments, biofilms interact with and compete against other microbial species for space and nutrients. Some of the metabolic alterations that we observed during biofilm development, such as pulcherriminic acid and antibiotic synthesis production, appeared related to bacterial competition. Starting at around 16 to 20 h in biofilm development, we observed extracellular accumulation of cLL and the iron chelator pulcherriminic acid, which combine with Fe^3+^ to form pulcherrimin (64, 65) (Fig. 8A and B). Consistent with the accumulation of these two metabolites, we observed dramatically increased transcript and protein levels of the pulcherriminic acid biosynthetic operon *yvmC-cypX* (Fig. 8C). Pulcherriminic acid production has been found in other *Bacillus* species and yeast (66–69). Although its role in biofilm development remains poorly understood, it is possible that upregulation of pulcherriminic acid biosynthesis may serve to sequester environmental iron to decrease the growth of surrounding competing bacterial species (64, 65, 70–72).

Alongside upregulation of pulcherrimin synthesis, we observed upregulation of the biosynthetic genes of two different antibiotics, namely, bacilysin and subtilosin A, encoded respectively by the *bacABCDEF* (73) and *sbo-alb* operons, the latter of which also confers immunity against subtilosin A (Fig. 8D and E). Subtilosin A is an antimicrobial peptide with activity against Gram-positive species such as Listeria monocytogenes (74, 75), while bacilysin is a nonribosomal peptide antibiotic active against a wide range of bacteria and some fungi (76).

### Transition from fatty acid synthesis to degradation during biofilm development.

**FIG 9:**
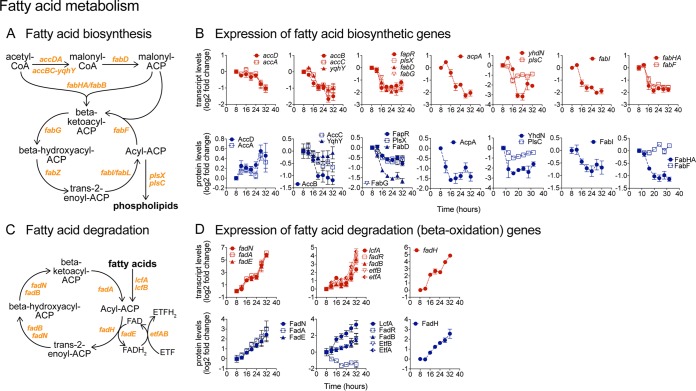
Shift from fatty acid synthesis to β-oxidation during biofilm development. (A) Fatty acid biosynthetic pathway. (B) Transcript and protein levels of fatty acid biosynthetic genes displayed as log_2_ fold changes relative to the initial 8-h time point. Genes within the same operon are shown in the same graph. Data represent averages of results from 4 (transcript) or 3 (protein) biological replicates ± SEM. (C) Fatty acid degradation pathways via β-oxidation. (D) Transcript and protein levels of fatty acid β-oxidation genes displayed as log_2_ fold changes relative to the initial 8-h time point. Genes within the same operon are shown in the same graph. Data represent averages of results from 4 (transcript) or 3 (protein) biological replicates ± SEM.

Fatty acid synthesis and degradation displayed striking alterations during biofilm development. The expression of most fatty acid biosynthetic genes, and their corresponding enzyme abundances, decreased markedly between 12 and 16 h (Fig. 9A and B). This included the concerted downregulation of the *accB-accC-yqhY* and *fapR-plsX*-*fabD-fabG-acpA* operons, the *fabI* gene, and the *fabHA-fabF* operon, encoding fatty acid biosynthetic enzymes such as acetyl-CoA carboxylase (AccB and AccC) (77), phosphate acyltransferase (PlsX), transacylase (FabD), reductase (FabG and FabI), and acyl carrier protein synthase (FabHB and FabF) (78).

Following a trend opposite that seen with fatty acid synthesis, transcript and enzyme levels in fatty acid β-oxidation displayed highly coordinated upregulation starting at around 16 h (Fig. 9C and D). The upregulated genes and operons included *lcfA-fadRB-etfBA*, *fadNAE*, and *fadH*, encoding fatty acid degradation enzymes such as long-chain acyl-CoA synthetase (LcfA), acyl-CoA dehydrogenase (FadN, FadE) and acyltransferase (FadA), and dehydratase (FadB) (79). LcfA is also involved in production of surfactin, which has been implicated as a potential signaling molecule in biofilm formation (80). The upregulation of fatty acid β-oxidation genes was in general of greater magnitude than the upregulation observed in fatty acid biosynthesis. These drastic alterations in fatty acid synthesis and degradation suggest potential remodeling of the cell membrane during biofilm development.

### Identification of transcriptional regulators involved in metabolic remodeling during biofilm development.

**FIG 10:**
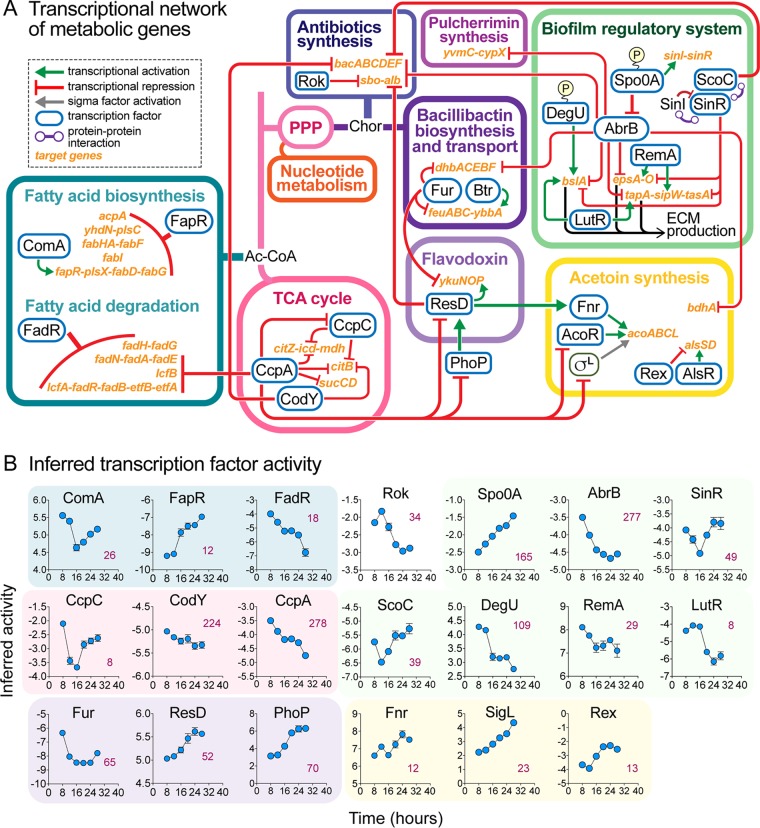
Transcriptional regulators involved in metabolic remodeling during biofilm development. (A) Transcriptional network of metabolic genes highlighted in this study. TCA cycle enzyme genes are transcriptionally regulated by the transcription factors CcpA, CcpC, and CodY. Together with FadR, CcpA also represses transcription of fatty acid degradation genes. Transcription of fatty acid biosynthetic genes is repressed by FapR, while the *fapR* operon is transcriptionally activated by ComA. CcpA also represses expression of transcription factors that regulate flavodoxin biosynthesis (ResD and PhoP) and acetoin biosynthesis (AcoR and SigL) genes (see also Fig. 12E). Flavodoxin genes (*ykuNOP* operon) are transcriptionally activated by ResD and repressed by Fur, which also represses expression of genes involved in bacillibactin biosynthesis (*dhbACEBF*) and transport (*feuABC-ybbA*)—the latter is also transcriptionally activated by Btr. ResD also represses expression of antibiotic synthetic genes (*sbo-alb* operon), which are also repressed by Rok and the transition state regulator AbrB. Another antibiotic synthetic operon, *bacABCDEF*, encoding bacilysin synthesis, is repressed by CodY and two biofilm and transition state regulators, AbrB and ScoC. AbrB represses expression of genes involved in pulcherrimin synthesis (*yvmC-cypX*) as well as expression of the extracellular matrix operons *epsA-O*, *bslA*, and *tapA-sipW-tasA*. AbrB is in turn transcriptionally repressed by Spo0A. Abbreviations: Ac-CoA, acetyl coenzyme A; Chor, chorismate. (B) Inferred transcription factor (TF) activity. The activity of TFs was inferred from transcriptome data using a published method (81). TFs involved in the same metabolic pathways are grouped together, with the background colors corresponding to those defined in the key in panel A.

The close agreement between changes in metabolite levels, transcript levels, and enzyme abundance that we observed in numerous pathways indicated that metabolic remodeling during biofilm development was in large part controlled at the transcriptional level. To identify the transcriptional regulators likely responsible for this metabolic remodeling, we performed a global analysis of transcription factor (TF) activity based on our gene expression data by applying network component analysis in conjunction with recently published models of the B. subtilis global transcriptional network (81, 82). Among 227 B. subtilis TFs, we identified 147 TFs with significant changes in activity (*P* < 0.001 with FDR = 0.00065) during biofilm development (see Table S2B at https://bit.ly/2Dn2HX2). This analysis recapitulated the expected behavior of TFs within the core biofilm regulatory network while providing a detailed picture of their temporal behaviors (Fig. 10). Specifically, the inferred activity of Spo0A increased steadily over the course of biofilm development, in agreement with decreasing *abrB* transcript levels and increasing *sinI* transcript levels (Fig. 10B; see also Fig. S5A). Also, as expected, the inferred activity of the biofilm repressor AbrB decreased rapidly in early biofilm development. In addition, SinR activity dropped early in biofilm development (8 to 16 h), as anticipated, before increasing back to initial levels after 24 h of growth.

10.1128/mBio.00623-19.5 FIG S5 Alternative utilization of sigma factors during biofilm development. (A) Transcriptional expression of the *abrB* and *sinI* biofilm regulatory genes displayed as log_2_ fold changes relative to the initial 8-h time point. Data represent averages of results from 4 biological replicates ± SEM. (B) Transcriptional network of sigma factor genes. (C) Inferred activity of selected B. subtilis sigma factors during biofilm development. Bars represent SEM of results from 4 biological replicates. Numbers shown in magenta indicate numbers of target genes corresponding to each sigma factor. (D) Transcript and protein levels of sigma factor genes displayed as log_2_ fold changes relative to the initial 8-h time point. Data represent averages of results from 4 (transcript) or 3 (protein) biological replicates ± SEM. (E) Biofilm development of *ΔsigL*, *ΔsigE*, and *ΔsigG* sigma factor deletion mutants compared to WT. (F) Spore fractions (percent) in WT strain versus *ΔsigE*, and *ΔsigG* mutants at 12, 24, and 32 h of growth. Download FIG S5, TIF file, 31.5 MB. Copyright © 2019 Pisithkul et al. 2019 Pisithkul et al. This content is distributed under the terms of the Creative Commons Attribution 4.0 International license.

Beyond the core biofilm regulatory network, our TF activity analysis identified transcriptional regulators likely responsible for the metabolic remodeling during biofilm development that we have described. The transcriptional network of metabolic genes highlighted in this study and the inferred activity of the transcription factors involved are presented in Fig. 10. We highlight some of the major conclusions below.

### (i) TCA cycle.

Upregulation of TCA cycle genes (*citZ-icd-mdh*, *citB*, and *sucCD*) during early biofilm development appeared to be driven by the simultaneous decreases in activity of the carbon catabolite control proteins CcpA and CcpC and the pleiotropic global regulator CodY, all three of which negatively regulate expression of genes in this pathway (Fig. 10; see also Fig. 4).

### (ii) ECM genes.

Transcription of ECM genes (*epsA-O*, *tapA-sipW-tasA*, and *bslA*) is controlled by core components of the biofilm regulatory network. Their initial increase in transcription was likely driven by increased Spo0A activity and the resulting decrease in activity of the transcriptional repressors AbrB and SinR. Their decrease in transcript levels at later time points may have been due to the recovery of SinR activity and decreased activity of transcriptional activators such as RemA, LutR, and DegU (Fig. 10).

### (iii) Iron metabolism.

The increased expression of bacillibactin synthesis genes (*dhbACEBF*) at the beginning of biofilm formation was likely driven by the simultaneous decrease in activity of the transcriptional repressors Fur and AbrB (83, 84). The concomitant increase in expression of iron-bound bacillibactin transporter genes (*feuABC*) may be similarly explained by decreased Fur activity together with increased Btr activity. Decreased Fur activity was also likely responsible for the upregulation of flavodoxin genes (*ykuNOP*), whose expression profile is nearly identical to that of bacillibactin synthesis and transport genes. Increased expression of pulcherrimin synthesis (*yvmC-cypX*) and subtilosin (*sbo-alb*) genes was likely driven by decreased activity of AbrB, while the transient upregulation of bacilysin biosynthetic genes (*bac* operon) was likely driven by the transient decrease in ScoC activity (Fig. 10; see also Fig. 8).

### (iv) Fatty acid metabolism.

The switch-like transition from fatty acid and phospholipid biosynthesis to fatty acid beta-oxidation that occurred between 12 and 16 h during biofilm development was likely driven by simultaneous but opposite changes in the activity of the transcriptional repressors FapR and FadR. FapR, whose inferred activity increased sharply between 12 and 16 h, represses expression of multiple operons encoding fatty acid biosynthetic genes. FadR, whose inferred activity decreased steadily over time, is a general repressor of fatty acid beta-oxidation pathways. Increased fatty acid degradation was also likely driven by decreased CcpA activity, while the decreased activity of the positive regulator ComA (85) may have also contributed to decreased fatty acid synthesis.

### (v) A central role for CcpA during biofilm development.

Some transcription factors displayed a global role in regulating metabolic remodeling during biofilm development. Specifically, our results indicate that the global metabolic regulator CcpA, which governs transcription of over 250 genes, plays an important role in regulating the TCA cycle, fatty acid beta-oxidation, and acetoin production during biofilm development. This agrees with a previous transcriptional study that reported that B. subtilis biofilm formation is catabolite repressed by CcpA (86).

### (vi) Sigma factor activity.

Finally, our TF activity analysis also indicated differential utilization of sigma factors during biofilm development. Specifically, the inferred activity of four sigma factors, σ^E^, σ^L^, σ^B^, and σ^D^, changed substantially (Fig. S5B and C). The activity of both σ^E^ and σ^L^ increased over time, σ^B^ activity decreased sharply between 12 and 20 h, and σ^D^ activity decreased gradually. Transcript levels of these four sigma factors closely followed their activity profiles, and, with the exception of σ^L^, their protein abundance was also well correlated with their activity (Fig. S5C and D). σ^D^ regulates flagella, motility, chemotaxis, and autolysis (87). The decrease in σ^D^ activity paralleled the decrease in abundance of flagellar and motility proteins such as flagellar basal-body rod protein FlgB, flagellin polymerization protein FliD, flagellin protein Hag, cell separase LytF, and autolysin LytC (Fig. S3F). σ^E^ is known to regulate transcription of sporulation genes in early mother cells (88, 89). σ^F^ and σ^G^ also regulate sporulation and, similarly to σ^E^, displayed a sharp increase in activity at 32 h. The increase in the activity of these sporulation-specific sigma factors paralleled the sharp rise in the levels of spore fractions during late biofilm development (Fig. 1D). σ^B^ activates numerous genes involved in stress response (90), and σ^L^ impacts carbon and nitrogen metabolism (91–93). As illustrated in Fig. S5B, transcription of *sigE* is activated by Spo0A and repressed by AbrB and SinR; therefore, its activity profile agreed with that of their regulators. The same was true for *sigD* and *sigL*, which are negatively regulated by Spo0A and CcpA, respectively. Interestingly, although *sigB* expression is also repressed by CcpA (94), it displayed an activity profile opposite that seen with σ^L^, indicating that other factors modulate its activity.

### Acetoin biosynthesis is crucial for robust B. subtilis biofilm growth.

### Upregulation of acetoin and 2,3-butanediol biosynthesis in early biofilm development.

**FIG 11:**
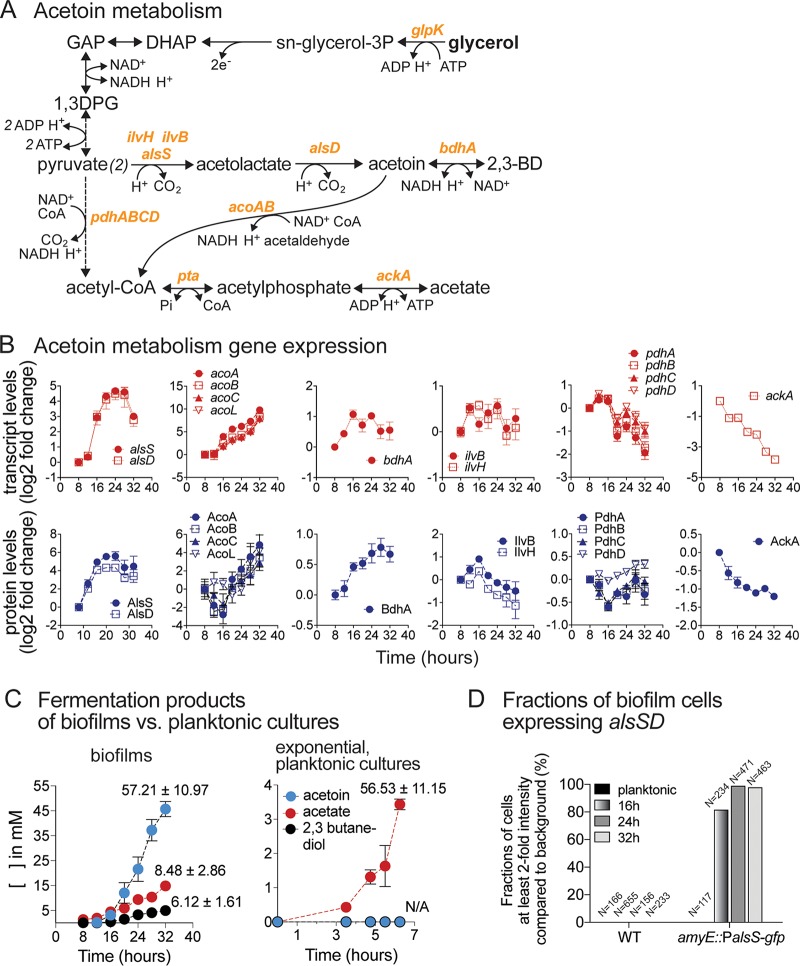
Upregulation of acetoin biosynthesis during biofilm development. (A) Acetoin and acetate fermentation pathways. Abbreviations: GAP, glyceraldehyde-3-phosphate; 1,3DPG, 1,3-diphosphoglycerol; 2,3BD, 2,3-butanediol. Enzyme genes are shown in orange text. (B) Transcript and protein levels of acetoin and acetate metabolism genes. Data are displayed as log_2_ fold changes relative to the initial 8-h time point. Genes within the same operon are shown in the same graph. Data represent averages of results from 4 (transcript) or 3 (protein) biological replicates ± SEM. (C) Production of major fermentation products (acetoin, acetate, and 2,3-butanediol) during biofilm development and in exponential-phase planktonic cultures. Data represent averages of results from 6 biological replicates ± SEM. (D) Fractions of cells actively expressing the *alsSD* operon. Promoter regions of *alsSD* were fused with *gfp*(*sf*) and integrated at the *amyE* locus to allow estimation of *alsSD* expression levels. Cells from planktonic cultures and 16 h, 24 h, and 32 h biofilms were observed under a fluorescence microscope. The GFP intensity of each object (cell) was recorded. The bar graph shows the fractions of cells with at least 2-fold-higher intensity than the background (see also Fig. S8).

To exemplify the biological relevance of the results obtained from our systems-level analysis of B. subtilis biofilm development, we performed a more extensively in-depth investigation of changes to acetoin metabolism. B. subtilis can produce acetoin from pyruvate in multiple ways. As shown in Fig. 11A, pyruvate may be converted to acetolactate by acetolactate synthase (IlvBH) in the branched-chain amino acid synthesis pathway or by a paralogous acetolactate synthase, AlsS. Acetolactate is then converted to acetoin by acetolactate decarboxylase (AlsD). Acetoin can then be reduced to 2,3-butanediol (2,3BD) by 2,3-BD dehydrogenase (BdhA). After its production, acetoin may be catabolized to acetyl-CoA and acetaldehyde by the acetoin dehydrogenase complex encoded by the *acoABCL* operon. In parallel to acetoin production, pyruvate may be metabolized into acetate via the combined action of the pyruvate dehydrogenase complex (PdhABCD), phosphotransacetylase (Pta), and acetate kinase (AckA) (Fig. 11A).

The upregulation of acetoin biosynthetic genes (*alsSD* operon) and acetoin utilization genes (*acoABCL* operon) was among the most significant alterations in gene expression seen during biofilm development (Fig. 11B). *alsDS* transcript levels increased sharply in early biofilm development (16 h), reaching a peak at 24 to 28 h (∼25-fold over initial levels) before decreasing somewhat at 32 h. *acoABCL* transcript levels remained low during early biofilm development (8 to 12 h) but increased gradually starting at 16 h to reach high levels at 32 h (>250-fold over initial levels). Transcriptional changes in the *alsSD* and *acoABCL* operons matched changes in their corresponding protein levels (Fig. 11B). In addition, BdhA protein levels also increased significantly during biofilm development, albeit to a lesser extent. Contrasting with the results seen with the acetoin and 2,3BD biosynthesis genes, transcripts and protein levels of AckA, the final step in acetate production, decreased during biofilm development. Protein levels of *ilvBH* displayed a small increase at 16 h but decreased afterwards, suggesting that acetolactate was produced primarily via AlsS at subsequent time points (Fig. 11B).

These observations prompted us to investigate acetoin production during biofilm development. Using ^1^H nuclear magnetic resonance (NMR) analysis, we observed significant acetoin production starting at ∼16 h, which corresponded with the timing of the sharp increase in transcript and protein levels of *alsSD* acetoin biosynthesis genes (Fig. 11C). Acetoin was the major fermentative product during biofilm development, reaching an extracellular concentration of ∼46 mM by 32 h, three times that of acetate (∼15 mM), the second-most-abundant product. Concurrently with acetoin production, we also observed production of 2,3BD (∼5 mM). Using ^13^C-glycerol, we found that these three fermentation products were produced exclusively from glycerol, with no measurable contribution from glutamate (Fig. S6). Acetoin synthesis during biofilm development corresponded to ∼57% of the glycerol consumed, while acetate and butanediol represented an additional ∼8% and an additional ∼6%, respectively. The large fraction of glycerol (∼70%) converted to these products indicated predominantly fermentative metabolism taking place during biofilm development (Fig. 11C). Interestingly, this was consistent with our observation that the media became anaerobic shortly after biofilm pellicle formation (∼16 to 20 h) (Fig. S7). Importantly, we found that acetoin and 2,3BD production were specific to biofilm development; we did not observe any measurable production (> 0.05 mM) of either metabolite during the exponential-phase growth, during which acetate was the main fermentation product (Fig. 11C).

10.1128/mBio.00623-19.6 FIG S6 Acetoin is produced exclusively from glycerol. (A) Heteronuclear single quantum coherence spectroscopy (HSQC) results showing production of ^13^C-labeled acetoin (red circles in the bottom panel) in spent media of biofilm cells grown on ^13^C-labeled glycerol. The appearance of acetoin (as well as of acetate and 2,3-butanediol) in the 2-dimensional (2D) spectrum of spent media of biofilm cells grown on ^13^C-labeled glycerol indicates that the compound is ^13^C-labeled. As a control, the top panel shows 2D spectra of ^12^C standard compounds (i.e., acetoin, acetate, and 2,3-butanediol) and the absence of ^13^C-labeled acetoin signal. (B) ^1^H NMR spectra of spent media of B. subtilis biofilms. The top spectrum shows chemical shift of acetoin, acetate, and 2,3-butanediol using standard compounds. The middle and bottom spectra show the differences between ^13^C-glycerol-treated and non-^13^C-glycerol-treated 32-h samples. The data show that >95% of the acetoin was ^13^C-labeled when cells were grown in ^13^C-glycerol. Download FIG S6, TIF file, 82.5 MB. Copyright © 2019 Pisithkul et al. 2019 Pisithkul et al. This content is distributed under the terms of the Creative Commons Attribution 4.0 International license.

10.1128/mBio.00623-19.7 FIG S7 Anaerobic environment underneath the pellicle biofilms. To determine anaerobic conditions in media underneath the pellicles, we performed a series of assays using resazurin as an oxygen indicator. (A) B. subtilis NCIB3610 WT, *ΔalsD*, and *ΔalsR* biofilms were grown in MSgg medium with resazurin for 20 and 24 h at 37°C. Cell-free MSgg medium with resazurin had blue-purple coloration. Anaerobic medium conditions are evidenced by the disappearance of resazurin coloration. (B) Color disappearance seen with resazurin added to MSgg in the presence of biofilm pellicles. Resazurin-added MSgg was blue at time *t* = 0. During B. subtilis biofilm growth (16-h and 20-h biofilms are shown), the blue coloring of resazurin disappeared. The media quickly regained a pink coloration after brief aeration, indicating that the loss in coloration was not due to degradation of resazurin over time and that resazurin remained a viable oxygen indicator and was visible against the background coloration produced by growing biofilms. (C) Side views of biofilm cultures showing that the pink coloration of aerated resazurin is easily visible against the natural color of spent medium of biofilm (- resazurin control). (D) The top panels show 20-h biofilms and noninoculated MSgg media controls used for this assay. The entire 12-well plate was transferred into an anaerobic chamber (Coy Laboratory) with an atmosphere of 90% nitrogen, 5% hydrogen, and 5% carbon dioxide. Subsequently, the supernatants (spent media) of the biofilm and the noninoculated medium control were transferred into another 12-well plate anaerobically prefilled with colorless resazurin solution (made anaerobic by storage in the anaerobic environment for several days prior to the experiment). Upon introduction of the 20-h-biofilm supernatant into the colorless resazurin, the mixture remained colorless (excluding the coloring of the spent medium). In contrast, introduction of noninoculated control MSgg media caused the previously colorless resazurin to immediately turn pink (middle panel). Therefore, the resazurin results indicated the absence of oxygen in spent biofilm media (no color change) and the presence of oxygen in the noninoculated MSgg medium controls. Finally, as shown in the bottom panel, the 12-well plate was removed from the environmental chamber and reexposed to oxygen. Resazurin mixed with the biofilm spent medium readily regained color, indicating the viability of the use of resazurin as an oxygen indicator. Download FIG S7, TIF file, 48.1 MB. Copyright © 2019 Pisithkul et al. 2019 Pisithkul et al. This content is distributed under the terms of the Creative Commons Attribution 4.0 International license.

Finally, using a fluorescent transcriptional reporter, we found that ∼80% of the cells within the biofilm displayed significant upregulation of the *alsSD* operon by 16 h of biofilm growth. By 24 and 32 h, >90% of the cells displayed strong (albeit variable) upregulation of the *alsSD* operon (Fig. 11D; see also Fig. S8). These data indicated that the majority of cells within the biofilm contributed to acetoin production.

10.1128/mBio.00623-19.8 FIG S8 Expression of *alsSD* in B. subtilis biofilms. (A) Expression of *alsSD* in individual cells from B. subtilis biofilms using a fluorescent transcriptional reporter (*amyE*::P*alsS-gfp*) (see Materials and Methods). Pictures taken under bright-field (BF) and GFP cube filters show the populations that expressed *alsSD*. The WT control shows no fluorescence background. (B) Summary of fractions of cells in planktonic cultures or biofilms that express the *alsSD* operon. Expression of this operon was measured using the intensity of GFP translated from *gfp* fused with promoter regions of *alsSD* integrated into the B. subtilis genome at the *amyE* locus. (C) Violin plots summarizing GFP intensities of WT and *amyE*::P*alsS-gfp* cells in exponential-phase planktonic cultures and 16-h, 24-h, and 32-h biofilms. Download FIG S8, TIF file, 70.1 MB. Copyright © 2019 Pisithkul et al. 2019 Pisithkul et al. This content is distributed under the terms of the Creative Commons Attribution 4.0 International license.

### Biofilm development is severely impaired in knockout strains defective in acetoin production.

**FIG 12:**
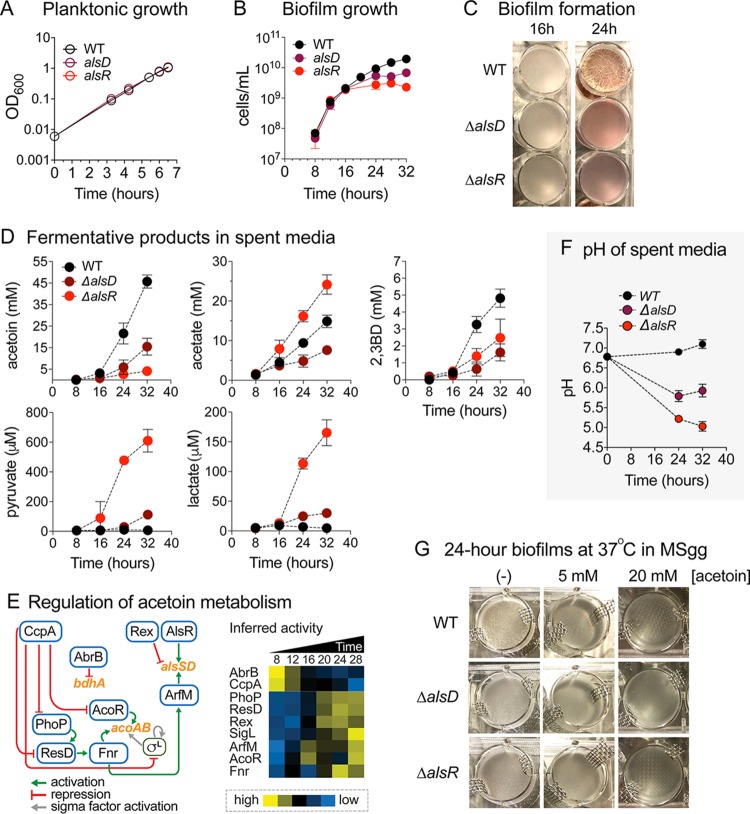
Acetoin biosynthesis is required for robust biofilm growth. (A) The exponential-phase growth (OD_600_) of *ΔalsD* and *ΔalsR* mutants was indistinguishable from that of the WT. Data represent averages of results from 3 biological replicates ± SEM. (B and C) Biofilm development was severely impaired with respect to growth (B) and formation (C) in *ΔalsD* and *ΔalsR* mutants after 16 h in terms of overall cell growth (cells per milliliter), pellicle thickness, and development of intricate biofilm structures. Data represent averages of results from 3 biological replicates ± SEM. (D) Fermentation products of spent media. Spent media of WT, *ΔalsD*, and *ΔalsR* biofilms at 8, 16, 24, and 32 h of growth were subjected to ^1^H NMR analysis. Data represent averages of results from 6 biological replicates ± SEM. (E) Regulation of acetoin metabolism. Acetoin biosynthetic genes *alsS-alsD* and *acoA-acoB* are regulated by interconnected webs of transcription factors, including AlsR, Rex, ArfM, Fnr, AcoR, SigL, and CcpA. 2,3-Butanediol biosynthetic gene *bdhA* is transcriptionally repressed by AbrB. The heat map on the right shows temporal inferred activities of transcription factors involved in the pathway. Yellow, high activity; blue, low activity. (F) pH of spent media. Line graph shows pH of WT, *ΔalsD*, and *ΔalsR* biofilm media at 24 and 32 h of growth. Data represent averages of results from 4 biological replicates ± SEM. (G) Acetoin addition to MSgg medium did not enhance biofilm growth of B. subtilis WT, *ΔalsD*, or *ΔalsR.* B. subtilis WT, *ΔalsD*, and *ΔalsR* were grown in MSgg medium with 0, 5, or 20 mM acetoin. Pictures show the growth level under each set of conditions after 24 h of incubation at 37°C.

To investigate the physiological relevance of the upregulation of acetoin biosynthesis during biofilm development, we created strains with impaired acetoin production by deleting *alsD* or *alsR*, each of which encodes a transcription factor that positively regulates expression of the *alsSD* operon (95). The exponential-phase growth of *ΔalsD* and *ΔalsR* mutants was indistinguishable from that seen with the wild-type (WT) strain (Fig. 12A), and biofilm development in the mutants up to 16 h was comparable to WT biofilm development as well. However, biofilm development was severely impaired in terms of overall cell growth, pellicle thickness, and development of intricate features after 16 h (Fig. 12B and C), which coincided with the timing of increased transcript and protein levels of *alsSD* in the WT strain and the approximated timing at which the growth media became anaerobic (Fig. S7). The *ΔalsD* and *ΔalsR* mutants produced significantly less acetoin than the wild-type strain, and overall 2,3BD production also decreased (Fig. 12D). The *ΔalsR* mutant also displayed significantly enhanced acetate production, accumulating to up to ∼25 mM versus ∼15 mM in the WT strain, which indicated carbon overflow from pyruvate into acetate. On a per-cell basis, both the *ΔalsD* and *ΔalsR* mutants displayed elevated acetate production. Interestingly, the *ΔalsR* mutant, which displayed the lowest level of acetoin production, had higher 2,3BD production than the WT strain on a per-cell basis and its ratio of acetoin to butanediol production shifted from approximately 9:1 in the WT strain to 2:1 in the *ΔalsR* mutant. This suggests that although less acetoin was produced, a larger fraction of it was reduced to butanediol.

Using LC-MS, we also observed extracellular accumulation of pyruvate and lactate in both the *ΔalsD* and *ΔalsR* mutants (Fig. 12D). The accumulation of pyruvate and lactate, in addition to increased acetate production, is consistent with blockage of acetoin production and redirection of pyruvate toward these alternative fermentative pathways. Intracellular metabolite measurements in the *ΔalsD* and *ΔalsR* mutants were also consistent with pyruvate overflow to other pathways (Fig. S9A and B). Specifically, we observed increased intracellular levels of alanine (produced from pyruvate), acetyl-phosphate (produced from acetyl-CoA), and *N*-acetylated amino acids (which use acetyl-phosphate as a biosynthetic precursor [96]); many of these metabolites were also secreted into the media.

10.1128/mBio.00623-19.9 FIG S9 Selected intracellular and extracellular metabolite levels of *ΔalsD* and *ΔalsR* mutant biofilms and the role of BdhA during biofilm development. (A) Intracellular metabolite levels of acetoin mutants during biofilm growth. The line graphs show relative metabolite levels of the *ΔalsD* and *ΔalsR* acetoin-deficient mutants compared to the WT at the corresponding time points, i.e., at 8, 16, 24, and 32 h of growth. Data represent averages of results from 3 biological replicates ± SEM. (B) Relative extracellular metabolite levels. The heat map displays log_2_ standard scores (z-scores) of individual extracellular metabolites over time (measurements taken at 8, 16, 24, and 32 h of growth). Yellow and blue shading indicates high and low metabolite levels, respectively. (C) The *ΔbdhA* and *ΔacoR* deletion mutants had robust biofilms compared to the WT. Shown are 24-h biofilms. (D) Fermentation products in *ΔbdhA* mutants measured by ^1^H NMR. Data represent averages of results from 4 biological replicates ± SEM. (E) Production of 2,3BD from extracellular acetoin. B. subtilis NCIB3610 pellicle biofilms were grown in MSgg at 37°C for 24 h. The 24-h biofilms were transferred (using stainless-steel mesh) to either the wells filled with 4.5 ml of MSgg medium (control) or those filled with 4.5 ml of MSgg medium supplemented with 20 mM acetoin (treatment). The samples were collected prior to the transfer and at 1, 4, and 8 h after the biofilms were transferred to the new wells. Samples were collected from the no-cell control as well to establish the baseline for acetoin, acetate, and 2,3BD concentrations in the incubated MSgg medium supplemented with 20 mM acetoin. These background concentrations in the treatment medium were shown in dashed lines ([2,3BD] = 0 mM). The quantification of acetoin, acetate, and 2,3BD was achieved by NMR analysis. (F) Changes in pH of MSgg medium upon acetoin or acetic acid addition. pH was monitored as up to ∼15 mM acetoin (blue dots) or acetic acid (red dots) was added to MSgg medium. Data represent averages of results from 2 independent replicates. Download FIG S9, TIF file, 14.2 MB. Copyright © 2019 Pisithkul et al. 2019 Pisithkul et al. This content is distributed under the terms of the Creative Commons Attribution 4.0 International license.

We constructed a *ΔbdhA* mutant to examine the extent to which the loss of acetoin-to-2,3BD conversion affected biofilm growth. However, the *bdhA* deletion did not significantly decrease production of butanediol or of any other measured fermentation products (i.e., acetoin or acetate) and therefore had no discernible effect on biofilm pellicle formation and development (Fig. S9C and D). This result suggested that there exists a redundant, unidentified butanediol dehydrogenase in B. subtilis NCIB3610 (in agreement with data reported in reference 97) but yielded no information on the role of butanediol production during biofilm development. Nevertheless, the observation that the fraction of acetoin converted to 2,3BD in the *ΔalsR* mutant had increased considerably compared to the WT strain provides evidence of the relevance of 2,3BD production for pellicle development. We also observed that extracellular acetoin present in the media can be reduced to 2,3BD (Fig. S9E), which suggests that cells could potentially use acetoin/2,3BD as an external electron acceptor/donor pair traveling between the anaerobic cells in the lower region of the pellicles and those at the aerobic top region. Nevertheless, supplementation of media with acetoin and/or 2,3BD (from time *t* = 0) did not rescue the defective biofilm development of the *ΔalsD* and *ΔalsR* mutants (Fig. 12G), which is consistent with our conclusion that the process of acetoin biosynthesis itself, and not the extracellular accumulation of acetoin, is required for robust biofilm growth. Finally, deletion of *acoR*, the transcriptional activator of the *acoABCL* operon, did not appreciably impair biofilm growth, suggesting that, unlike acetoin biosynthesis, upregulation of acetoin degradation was not critical for biofilm development (Fig. S7C).

### Transcriptional regulation of acetoin metabolism genes during biofilm development.

The operons involved in acetoin metabolism (*alsSD*, *acoABCL*, *bdhA*, and *ilvBH*) are regulated by an interconnected web of transcription factors (Fig. 12E). In addition to AlsR, *alsSD* transcription is also activated by an uncharacterized transcription factor, ArfM (98, 99), itself activated by Fnr, a transcriptional regulator of anaerobic genes that also promotes transcription of *acoABCL*. *fnr* is transcriptionally activated by ResD, whose expression is in turn regulated by PhoP, a transcription factor that regulates over 70 genes involved in phosphate metabolism. Upstream of these transcription factors sits the carbon catabolite control protein CcpA, which directly represses *acoR*, *phoP*, *resD*, and *sigL* (σ^L^ is required for *acoABCL* transcription) (91). The inferred activity profile of these transcription factors is highly consistent with the observed upregulation of acetoin biosynthesis and utilization genes during biofilm development (Fig. 12E).

### Acetoin biosynthesis plays a role in maintaining extracellular pH.

Finally, we also observed that acetoin biosynthesis has a surprising and critical role in maintaining a neutral medium pH during biofilm development. By 32 h, the medium pH in the *ΔalsD* and *ΔalsR* mutants dropped from an initial ∼6.8 to ∼5.9 and ∼5.0, respectively, while in WT strain it increased slightly to ∼7.1 (Fig. 12F). The acetate produced in either the WT strain or the mutants was sufficient to acidify the media (Fig. S9F), but the fact that the pH did not decrease in WT biofilm indicates the presence of an active process maintaining or increasing the pH that is dependent on acetoin production. Pyruvate conversion to acetoin utilizes 2 protons (H^+^), and its reduction to butanediol requires an additional one (Fig. 11A), thereby constituting a mechanism for maintaining a neutral pH during biofilm development. However, increasing the buffer capacity of the media did not rescue the biofilm growth phenotype of the *ΔalsD* and *ΔalsR* mutants, indicating that the drop in pH was not solely responsible for their defective biofilm development.

## DISCUSSION

### Coordinated upregulation of energy-generating and biosynthetic pathways during early biofilm development.

Our integrated metabolomic-transcriptomic-proteomic studies revealed a widespread and dynamic remodeling of metabolism during B. subtilis biofilm development that affected central carbon metabolism, primary biosynthetic pathways, fermentation pathways, and secondary metabolism. Many of these metabolic alterations were previously unrecognized as being biofilm associated. A common theme of the metabolic remodeling during early biofilm development was an upregulation of energy-generating pathways (i.e., TCA cycle, glycerol catabolism) and biosynthetic pathways (e.g., nucleotides, amino acids, secondary metabolites, and ECM precursors). Upregulation of the TCA cycle may be particularly important in providing energy (i.e., ATP, GTP) and reducing power [i.e., NAD(P)H] to biosynthetic pathways during early biofilm development. The concerted upregulation of TCA cycle activity, nucleotide biosynthesis, and amino acid synthesis represents a coordinated response that may help support ECM production and other biofilm-specific processes. For example, we found that eDNA production, whose importance during early biofilm development has been previously demonstrated in numerous bacterial species (56, 57, 100), closely matched the transient increase in the levels of intracellular nucleotides and their biosynthetic precursors, suggesting that upregulation of nucleotide biosynthesis is required to support eDNA production. Additionally, high-level availability of nucleotide triphosphates and deoxynucleotides may help support the increased rates of mRNA and enzyme synthesis required for widespread metabolic remodeling during early biofilm development.

Interestingly, the fatty acid synthesis pathway was the only major biosynthetic pathway that displayed distinct downregulation in early biofilm development. Downregulation of fatty acid synthesis occurred concurrently with strong upregulation of fatty acid β-oxidation. These striking and contrasting alterations in fatty acid metabolism suggest that substantial remodeling of the lipid membrane composition may occur during biofilm development. A previous study in P. aeruginosa showed decreased levels of non-even-numbered chain phospholipids and increased levels of long-chain phosphatidylethanolamine in biofilms compared with planktonic cells (101). Similar changes may occur in B. subtilis since we observed significantly reduced expression of both *plsX* and *plsC*, which are involved in phospholipid biosynthesis (Fig. 9B). In addition, increased expression of long-chain acyl-CoA synthetase (*lcfA*) (Fig. 9D) and long-chain fatty acid-CoA ligase (*lcfB*) may lead to higher intracellular concentrations of long-chain acyl-CoA esters, which can act as regulatory (inhibitory) ligands for enzymes involved in lipid and energy metabolism (102, 103). Further research is required to investigate these hypotheses.

### Metabolic changes related to nutrient availability and transport: iron uptake and acetoin production.

Bacteria within pellicle biofilms are tightly associated with each other, and cells within different regions of the biofilm may experience distinct nutrient environments (38). Some of the metabolic alterations that took place during biofilm development, most notably, alterations in iron and acetoin metabolism, likely occurred to address challenges associated with transport or availability of specific nutrients.

### (i) Upregulation of iron uptake.

We observed strong concerted upregulation in the production of the siderophore bacillibactin and its corresponding ABC transporters during biofilm development that indicated a need for biofilm cells to improve iron assimilation (Fig. 7). The availability of extracellular iron (i.e., the concentration in the medium) did not decrease appreciably during biofilm growth (see Fig. S3E in the supplemental material), ruling out the possibility that the upregulation of iron uptake was due to long-term growth without nutrient replenishment. Instead, our observations suggest that upregulation of iron uptake is an essential aspect of B. subtilis biofilm development, likely resulting from the challenge of iron acquisition within the packed biofilm environment. The fact that the *dhb* operon, encoding bacillibactin biosynthetic genes, is negatively regulated by the core biofilm regulator AbrB provides further support to this hypothesis. Our observations also imply that bacillibactin, and its iron-bound form ferri-bacillibactin, can efficiently traverse the extracellular matrix to deliver iron to cells deep within the biofilm structure and to upper regions of the biofilm. Our findings are consistent with a recent study reporting that siderophore production is required to maintain iron homeostasis and robust biofilm growth in B. subtilis (104).

Although we hypothesize that upregulation of iron uptake systems results from challenges associated with iron transport within the biofilm, it is also possible that improved iron uptake helps increase the activity of specific metabolic pathways, such as the TCA cycle pathway, that involve iron-dependent enzymes (e.g., aconitase and succinate dehydrogenase). Interestingly, together with upregulation of iron uptake, we found that the iron-sulfur protein ferredoxin, which mediates electron transfer in numerous reactions, was replaced by iron-free flavodoxin as biofilm development progressed (Fig. 7G and H). This agrees with a previous study reporting that flavodoxin replaces ferredoxin under iron-limiting conditions (105). The switch in electron transfer proteins, from ferredoxin to flavodoxin, may have significant effects on the activity of a wide variety of enzymes. Specifically, in B. subtilis, flavodoxin serves as a redox partner for multiple enzymes such as nitric oxide-synthase (106), cytochrome P450 BioI (107), and the acyl lipid desaturase Des involved in fatty acid desaturation (108). In addition, ferredoxin is an abundant protein, and its replacement by flavodoxin may free iron for its utilization by other iron-dependent enzymes, helping maintain high metabolic activity in an iron-limited environment.

### (ii) Upregulation of acetoin production.

Another metabolic alteration likely related to nutrient (i.e., oxygen) availability was the upregulation of the acetoin fermentation pathway. Our results showed that acetoin production via AlsD was required for robust biofilm pellicle development and that the transcription factor AlsR played an essential role in controlling this process (Fig. 12). Upregulation of acetoin production during biofilm pellicle development was likely a response to the oxygen-limited environment that developed after early biofilm development. While cells at the pellicle surface were directly exposed to air and had access to abundant oxygen, those below the surface and at the bottom of the pellicle biofilm were likely to experience an oxygen-depleted environment (Fig. S7). We hypothesize that this phenomenon triggers the observed fermentative metabolism and the shift from acetate to acetoin and butanediol production that takes place in the transition from planktonic to biofilm growth. More specifically, conversion of glycerol to pyruvate yields one ATP and two NADH equivalents. The subsequent fermentation of pyruvate to acetoin is a redox-balanced process that yields no ATP. In contrast, fermentation of pyruvate to acetate produces one ATP along with one NADH equivalent (Fig. 11A). Although acetate fermentation would allow cells to maximize ATP production, acetoin production is expected to be favored under oxygen-limiting conditions where NAD^+^ regeneration from NADH becomes a limiting factor. It allows cells to produce ATP from conversion of glycerol to pyruvate, which can then be converted to acetoin without additional production of NADH equivalents. In addition, reduction of acetoin to 2,3BD, which consumes one NADH equivalent, can further help maintain redox balance in an oxygen-limited environment.

The metabolic alterations that arise in response to a heterogeneous nutrient environment may be confined to the specific cell subpopulations that are challenged by limited nutrient availability as determined by their location within the biofilm (38). Specifically, upregulation of bacillibactin production may be strongest in cells localized in the middle to upper regions of the pellicle, while acetoin production may be highest in cells localized in the middle to lower regions. Further experiments are required to test these hypotheses, but, in the case of acetoin, we found that >90% of the cells within the biofilm participated in its production (Fig. 11D; see also Fig. S8). A recent study found distinct B. subtilis subpopulations in exponential-phase cultures that either produced acetate or expressed acetoin biosynthetic genes (109) and reported that the acetoin-producing population became more prominent during stationary-phase growth. This is consistent with our observation of a metabolic switch from acetate to acetoin fermentation as biofilm development progressed and with our *alsSD* fluorescent transcriptional reporter data (Fig. 11D; see also Fig. S8).

### Metabolic alterations related to bacterial competition: pulcherriminic acid and antibiotic synthesis.

A subset of the metabolic alterations that we observed during biofilm development could have been related to bacterial competition in natural environments. For example, upregulation of pulcherriminic acid production may have the purpose of sequestering environmental iron to decrease the growth of surrounding competing bacterial species (110). However, a potential caveat is that excessive production of pulcherriminic acid can cause iron starvation for the producer cells (72). In this regard, the simultaneous production of bacillibactin and pulcherrimin appears contradictory since pulcherrimin may interfere with iron assimilation via bacillibactin. This apparent contradiction suggests that B. subtilis may have a mechanism for retrieving pulcherrimin-bound iron. Bacillibactin is a hexadentate catecholate siderophore that, together with enterobactin, has one of the strongest known affinities for iron (62, 111). Therefore, it is likely that the affinity of bacillibactin for iron is significantly stronger than that of pulcherriminic acid. If this were the case, pulcherriminic acid would decrease iron availability only for bacterial species lacking a high-affinity siderophore capable of stripping iron from pulcherrimin but it would not affect iron assimilation by bacillibactin-producing B. subtilis. Therefore, the concomitant upregulation of bacillibactin and pulcherriminic acid by B. subtilis may constitute an efficient iron uptake system in which pulcherriminic acid sequesters environmental iron without interfering with bacillibactin-dependent iron uptake.

Also related to bacterial competition, we observed upregulation of the antibiotics bacilysin, a nonribosomal peptide active against a wide range of bacteria and some fungi (76), and subtilosin A, a peptide with activity against Gram-positive species (74, 75). Production of these two antibiotics during biofilm growth may help B. subtilis compete against surrounding bacterial or fungal species.

### Secretion of TCA cycle intermediates and symbiosis with plant roots.

B. subtilis can establish a symbiotic relationship with plant roots by forming biofilms. This association is thought to be facilitated by plant root secretion of certain metabolites that recruit beneficial bacteria to form biofilms (23, 112). The TCA cycle intermediate malate has been identified as a plant root-secreted metabolite that promotes the development of B. subtilis biofilms (112). In this study, correlating with upregulation of TCA activity, we observed transient extracellular accumulation of malate and fumarate during early biofilm development (see Table S1C at https://bit.ly/2XrPH9Z). This observation agrees with the hypothesis that malate and, potentially, other TCA cycle intermediates serve as extracellular signals that potentiate biofilm growth. It has also been shown that the acetoin and 2,3-butanediol produced by B. subtilis GB03 and B. amyloliquefaciens IN937a promoted *Arabidopsis* growth (113). Therefore, in addition to the roles discussed previously, secretion of these two metabolites during B. subtilis biofilm development may also function to promote plant growth or act as a growth-promoting signal in natural environments.

### Fast dynamics of intracellular metabolites during early biofilm development.

Early biofilm development was characterized by fast and large changes in intracellular metabolite levels, which gave rise to the possibility of missing interesting dynamic behaviors using a 4-h sampling interval. However, metabolome profiles obtained with a sampling frequency of 1 h aligned well with the ones from our original experiment, indicating that a 4-h sampling interval captured all relevant metabolome dynamics (Fig. S10; see also Table S1D at https://bit.ly/2XrPH9Z).

10.1128/mBio.00623-19.10 FIG S10 B. subtilis metabolome during early-stage biofilm development. (A) Samples for LC-MS metabolomic analysis were taken every hour from h 10 to h 18 during biofilm development. Metabolite measurements were normalized by cell numbers and are displayed as log_2_ z-scores. The yellow (high) and blue (low) color scale indicates relative metabolite abundance levels across the time course. (B) Growth of B. subtilis in MSgg medium. B. subtilis cells were grown in standing cultures in a 12-well plate format. Growth was quantified by a direct cell count (cells per milliliter). Black dots represent means ± SEM of results from 4 to 8 biological replicates from samples collected in a 4-h interval. Blue dots represent means ± SEM of results from 3 biological replicates from samples collected in a 1-h interval. Download FIG S10, TIF file, 10.6 MB. Copyright © 2019 Pisithkul et al. 2019 Pisithkul et al. This content is distributed under the terms of the Creative Commons Attribution 4.0 International license.

### Transcriptional control of metabolism during biofilm development.

The regulation of metabolic activity occurs at different levels, including the transcriptional, translational, and posttranslational levels. For multiple pathways, we found excellent correlation between changes in metabolite levels and transcript levels and enzyme abundance, indicating that transcriptional regulation plays a central role in metabolic remodeling during biofilm development (Fig. 6; see also Fig. 10). In bacteria, fast-acting posttranslational regulatory mechanisms such as allosteric regulation and covalent protein modification regulate metabolism at short time scales (seconds to minutes) (114–116). This fast response correlates well with the expected need of bacteria to rapidly respond to fast environmental perturbations. Transcriptional regulation is a comparatively much slower form of regulation that represents a more extensively long-term commitment to metabolic remodeling. Therefore, without discarding the idea of a significant role for both allosteric regulation and posttranslational modifications, it seems logical for transcription to play a major role in regulating metabolism in the relatively slow (i.e., time scale of hours) developmental process of biofilm development. Although we observed an overall excellent correlation between transcript and protein levels (Fig. 3), we also identified several instances where translational regulation appeared to override transcription (see Table S3A at https://bit.ly/2XqQAzF). As an interesting example, the transcriptional repressor of fatty acid synthesis FadR displayed a large and statistically significant upregulation at the transcript level but its protein level was actually strongly downregulated (Fig. 9D), which contrasted with the rest of the genes in its operon, which showed excellent correlation between transcript and protein levels. Two additional examples of overriding translational regulation are provided by *purF* and *guaB*, involved in nucleotide biosynthesis (Fig. 5F).

Our analysis also identified the transcriptional regulators likely responsible for metabolic remodeling during biofilm development. These results expand the network of transcription factors involved in biofilm development beyond the core/classical transcriptional regulatory network controlling ECM, sporulation, and motility (Fig. 10). Finally, our TF activity analysis also indicated differential utilization of sigma factors during biofilm development. Specifically, the inferred activity of the sigma factors σ^E^, σ^L^, σ^B^, σ^D^, σ^G^, and σ^F^ changed significantly (Fig. S5C). σ^L^ has been shown to be required for biofilm formation in Burkholderia cenocepacia (117) and Vibrio fischeri (118). However, we found that deletion of σ^L^ did not cause any overt biofilm growth phenotype, suggesting that this sigma factor does not play an essential role in pellicle biofilm development in B. subtilis (Fig. S5E). Additionally, even though it virtually eliminated spore formation (Fig. S5F), deletion of σ^E^ or σ^G^ did not cause any obvious biofilm growth defect either (Fig. S5E), suggesting that neither of these two sporulation-specific sigma factors plays an essential role in pellicle biofilm development.

### Metabolic heterogeneity.

The present study was designed to investigate metabolism in B. subtilis pellicle biofilms taken as a complete entity. However, a significant advance in recent years has been the discovery of spatial and cell type heterogeneity within B. subtilis biofilms (119–123). It is likely that a subset of the metabolic alterations that we report here do not occur uniformly across the biofilm or take place only within specific cell subpopulations. We already noted potential heterogeneity arising from the cell subpopulations that upregulate iron uptake and acetoin production, but other metabolic alterations may also be unevenly distributed across the biofilm. For example, upregulation of TCA cycle and oxidative PPP activity may be predominant in upper regions of the biofilm that have preferential access to oxygen. By providing a detailed catalog of the genes, operons, and transcription factors contributing to major metabolic alterations during B. subtilis biofilm development, this work provides a unique foundation for future research aiming to quantitatively investigate spatiotemporal expression patterns of metabolic genes/operons at the single-cell level during biofilm growth.

### Conclusion.

This report presents a comprehensive systems-level investigation that has generated novel insights into the metabolic remodeling occurring during bacterial biofilm development and will serve as a unique hypothesis-generating resource for future studies on biofilm physiology that investigate the biological significance of the major metabolic alterations that we report in this study.

## MATERIALS AND METHODS

### Strain and media.

Bacillus subtilis strain NCIB3610 was maintained on Luria-Bertani (LB) agar plates. Floating biofilms (pellicles) were grown in modified MSgg medium (20) adjusted to pH 6.8 ± 0.05. The modified MSgg medium contains 2% (vol/vol) glycerol, 1% (wt/vol) glutamate, 5 mM potassium phosphate (pH 7), 5 mM MOPS (morpholinepropanesulfonic acid) (pH 7), 2 mM MgCl_2_, 700 μM CaCl_2_, 50 μM MnCl_2_, 50 μM FeCl_3_, 1 μM ZnCl_2_, and 2 μM thiamine.

### Cell handling and sample preparation (biofilm formation).

Pellicles were grown in a 12-well polystyrene plate format at 37°C. Each well contained a 4.5-ml culture volume with a custom-made stainless-steel mesh (Fig. 1F). Samples were collected every 4 h from 8 to 32 h after inoculation (in a 1:1,000 dilution) from a late-exponential-phase LB culture (OD_600_ of ∼1.0 to ∼1.5) into MSgg medium. To collect each sample, the stainless steel mesh was lifted (with intact pellicle on top of the mesh) and immediately submerged under 1.5 ml of −20°C cold extraction solvent (40:40:20 acetonitrile [ACN]/methanol/water). The pellicle was passed through a syringe needle (23-gauge; 0.6 mm by 25 mm) a total of 3 to 4 times to ensure exposure to the extraction solvent. For the 8-h (planktonic) sample, 25 ml of standing culture (6 wells combined) was rapidly filtered through 47-mm-diameter round hydrophilic nylon filters (Millipore catalog no. HNWP04700). The filter retaining the cells was immediately submerged in 1.5 ml of −20°C cold extraction solvent. The extract and cell debris were washed from the filter and transferred to a microcentrifuge tube. All extract mixtures were subjected to centrifugation at maximum speed (20,817 × *g*) for 5 min at 4°C. The supernatants (metabolome extracts) were stored at −20°C for analysis. The pellets were completely air-dried and weighed.

### Growth measurements.

Biofilm growth was quantified directly by cell count. Pellicles were dispersed using syringe needles. The resulting resuspensions were mildly sonicated (two 10-s pulses with a 45-s gap, 25% amplitude) (QSonica model Q125; Qsonica, Newtown, CT [125 watts, 20 kHz]) and fixed using 4% paraformaldehyde (124). The fixed cells were imaged under an Olympus IX83 inverted microscope using a 60× oil immersion objective. Images were taken using a Hamamatsu digital charge-coupled-device (CCD) camera and visualized using MetaMorph for Olympus software (Molecular Devices, Inc.). The resultant cells-per-milliliter values were used for normalization of metabolite levels. Spore fractions (percent) of biofilms were determined by dispersing biofilms with a syringe needle and heating them at 80°C for 20 min. Heated samples and nonheated control samples were subjected to serial dilution and spread onto LB agar plates followed by an overnight incubation at 37°C. Colonies from heated samples were compared with those from nonheated samples.

### Metabolite measurements.

Cell extract samples were dried under N_2_ and resuspended in LC-MS-grade water (Chromasolv; Sigma-Aldrich). Samples were analyzed using an ultra-high-pressure liquid chromatography-tandem mass spectrometry (UHPLC-MS/MS) system consisting of a Dionex UHPLC system coupled by electrospray ionization (ESI; negative polarity) to a hybrid quadrupole–high-resolution mass spectrometer (Q Exactive Orbitrap; Thermo Scientific) operated in full scan mode for detection of targeted compounds based on their accurate masses and retention times matched to purified standards. Properties of full MS-selected ion monitoring (MS-SIM) included a resolution value of 70,000, an automatic gain control (AGC) target value of 1 × 10^6^, maximum injection time (IT) of 40 ms, and a scan range of 70 to 1,000 *m/z*. Liquid chromatography (LC) separation was achieved using an Acquity UPLC BEH C_18_ column (Waters, Milford, MA) (2.1 by 100 mm, 1.7-μm particle size). Solvent A was 97:3 water/methanol with 10 mM tributylamine (TBA) adjusted to pH 8.1 to 8.2 with ∼9 mM acetic acid. Solvent B was 100% methanol. The total run time was 25 min with the following gradient: 0 min, 5% solvent B; 2.5 min, 5% solvent B; 5 min, 20% solvent B; 7.5 min, 20% solvent B; 13 min, 55% solvent B; 15.5 min, 95% solvent B; 18.5 min, 95% solvent B; 19 min, 5% solvent B; 25 min, 5% solvent B. The flow rate was 200 μl/min. Other LC parameters were as follows: autosampler temperature, 4°C; injection volume, 5 μl; column temperature, 25°C. Experimental MS data in the mzXML format were used for metabolite identification. Metabolite peaks were identified using MAVEN (metabolomics analysis and visualization engine) (125, 126). Metabolite quantitation was achieved using ^13^C-labeled Escherichia coli metabolites as internal normalization standards (described in the supplemental material). E. coli intracellular metabolite concentrations were obtained from previous studies (127).

### Use of ^13^C-labeled E. coli metabolites as internal normalization standards.

E. coli RL3000 (kindly given by Robert Landick, Bacteriology Department, University of Wisconsin [UW]—Madison) was maintained on an LB agar plate. A single colony was inoculated into M9 minimal medium with 0.4% (vol/vol) ^13^C-labeled glucose. The first labeled culture was incubated at 37°C overnight and inoculated into fresh M9 ^13^C-labeled glucose in a baffled flask (1:100 ratio) at 37°C with rigorous shaking. The culture (OD_600_ of ∼0.45) was extracted by rapid filtration through a 47-mm-diameter round hydrophilic nylon filter (Millipore catalog no. HNWP04700). The filter retaining the cells was immediately submerged (cells facing down) into 1.5 ml of −20°C cold extraction solvent. The extract and cell debris were washed from the filter and transferred to a microcentrifuge tube. All extract mixtures were subjected to centrifugation at maximum speed (20,817 × *g*) for 5 min at 4°C. The supernatants (metabolome extracts) were stored at −20°C until mixing and processing were performed with the biofilm metabolite extracts. Nonlabeled (^12^C) biofilm extracts were spiked with ^13^C-labeled E. coli extract and subjected to LC/MS analysis. The nonlabeled (^12^C) metabolite levels were normalized to the levels of the ^13^C-labeled counterparts. The resultant numbers were then normalized by cell growth as described above.

### Dynamic labeling experiments using ^13^C-glycerol.

B. subtilis NCIB3610 cells were grown in nonlabeled MSgg medium in the 12-well plate format (see Fig. 1). At 16, 24, and 32 h after initial growth, the pellicles were transferred to MSgg medium with ^13^C-labeled glycerol (Cambridge Isotope Laboratories, Inc., Andover, MA) to allow incorporation of ^13^C into biofilm cells for 0.5, 1, 2, 4, 7, and 10 min before the pellicles were submerged in the −20°C cold extraction solvent and processed for metabolite analysis as described above.

### RNA isolation.

Planktonic cells and/or pellicles (4 biological replicates per sample) were washed in −20°C cold methanol (50% [vol/vol] final concentration) and subjected to centrifugation. The supernatant was discarded, whereas the pellet was immediately freeze-dried using liquid nitrogen and stored at −80°C until extraction. To extract RNA, the pellet (volume of approximately 100 μl) was thawed on ice and resuspended in 200 μl of lysozyme–Tris-EDTA (TE; 10 mM Tris-Cl, pH 7.5; 1 mM EDTA, pH 8) by vortex mixing before a 20-min incubation at 37°C was performed. The RNA in the resultant mixture was extracted using an RNeasy minikit (Qiagen, Valencia, CA, USA) per the manufacturer’s instructions. Residual DNA was enzymatically removed using DNase (RNase-free DNase set; Qiagen).

### Construction of prokaryotic Illumina RNA libraries.

Total RNA submitted to the University of Wisconsin—Madison Biotechnology Center was verified for purity and integrity via the use of a NanoDrop 2000 Spectrophotometer and an Agilent 2100 BioAnalyzer, respectively. The samples that met the Illumina sample input guidelines were then prepared according to the instructions in the TruSeq RNA preparation guide. For each sample’s TruSeq RNA library preparation, 1 μg of total RNA was ribosomally reduced using a RiboZero rRNA removal (bacteria) kit (Illumina, San Diego, CA, USA). rRNA-depleted samples were purified by the use of Agencourt RNAClean XP beads (Beckman Coulter, Indianapolis, IN, USA). Subsequently, rRNA-depleted samples were fragmented using divalent cations under conditions of elevated temperature and immediately reverse transcribed into double-stranded (ds) dscDNA using SuperScript II reverse transcriptase (Invitrogen, Carlsbad, CA, USA) and random primers. The ds cDNA was purified by the use of paramagnetic beads, end-repaired by the use of T4 DNA polymerase and Klenow DNA polymerase, and phosphorylated by the use of T4 polynucleotide kinase. The blunt-ended cDNA was purified by the use of paramagnetic beads and then incubated with Klenow DNA polymerase to add an “A” base (adenine) to the 3′ end of the blunt phosphorylated DNA fragments. DNA fragments were ligated to Illumina adapters, which have a single “T” base (thymine) overhang at their 3’end. The adapter-ligated products were purified by the use of paramagnetic beads. Products of the ligation were subjected to PCR amplification using Phusion DNA polymerase and Illumina's genomic DNA primer set and were purified by the use of paramagnetic beads. The quality and quantity of the finished libraries were assessed using an Agilent DNA 1000 chip (Agilent Technologies, Inc., Santa Clara, CA, USA) and a Qubit dsDNA HS assay kit (Invitrogen, Carlsbad, CA, USA), respectively. Libraries were standardized to 2 μM. Cluster generation was performed using standard cluster kits (v3) and the Illumina cBot. Single-read 100-bp sequencing was performed by the use of standard SBS chemistry (v3) on an Illumina HiSeq 2000 sequencer. Images were analyzed using the standard Illumina Pipeline, version 1.8.2.

### RNA sequencing data analysis.

Using an Illumina Hiseq 2000 system, we obtained over 10,000,000 reads per library. The data from the Bacillus subtilis NCIB3610 chromosome (GenBank accession number CP020102.1) (52) and plasmid pBS32 (GenBank accession number NZ_CP020103.1) were concatenated into a single FASTA file. Single-end reads (100 bases) were aligned to the sequences in the resulting FASTA file using BWA-MEM version 0.7.15, with the following command line flags set: -t 8 -M (128). Alignment files were prepared using SAMtools, version 1.3.1 (129). The number of reads aligning to each coding sequence was determined using the “summarize overlaps” function from the R package *GenomicAlignments* (130), with the arguments *ignore.strand* = TRUE and mode = “*IntersectionNotEmpty*.” Read count normalization and calculation of differential expression levels were performed using a combination of the R package *edgeR* and the *voomWithQualityWeights* function from the R package *limma* (131, 132). Calculations of FDR data were performed by the method of Benjamini and Hochberg (133). We filtered the resultant transcriptome data on the basis of their minimum reads per kilobase per million (RPKM) values and the reproducibility across the four biological replicates (on the basis of the coefficients of variation [CVs]). Only the genes that passed this filter (3,690 open reading frames [ORFs]) were subjected to further analyses.

### Inferring transcription factor activities.

Data on inferred transcription factor activities were obtained using an established method (Inferelator [81]). The data on transcription factors with their target genes were retrieved from a gold standard in a publication that corresponded to the SubtiWiki database (134). The prior value was arbitrarily set to 1.6. The mRNA sequencing (mRNA-seq) data (input) were filtered by the average RPKM cutoff value (average RPKM of >25) and percent coefficient of variation (%CV of <60).

### NMR spectroscopy.

NMR samples contained 180 μl of growth medium, 20 μl of 5 mM dextran sulfate sodium (DSS), and D_2_O in 3-mm-diameter tubes. The spectra were acquired on a Bruker Avance III (500 MHz do ^1^H) equipped with a 5-mm-diameter triple-resonance inverse with carbon observe (TCI) cryogenic probe (Bruker, Billerica, MA). The water signal was suppressed by the excitation sculpting technique (zgesgp on Bruker Topspin). ^1^H spectra were acquired from 256 scans. Heteronuclear single quantum coherence spectroscopy (HSQC) spectra were acquired with 256 increments, 32 transients, and 2 s of relaxation delay. The ^1^H spectral width was 16 ppm, and the ^13^C spectral width was 175 ppm. The temperature was regulated at 298 K for all spectra. NMR was used for detection of fermentation products in the spent biofilm-inducing medium.

### Protein digestion and isolation.

Three biological replicates of B. subtilis samples were collected by centrifugation. The supernatant was discarded, and the pellet was freeze-dried using liquid nitrogen and stored at −80°C until submission.

The lysis method used for the B. subtilis pellet was modified from a previously described method (135). Each sample was suspended in 300 μl guanidine HCl (6 M) and heated for 2 cycles of 5 min at 100°C and was then subjected to 5 min of cooling at ambient temperature. Protein concentrations were determined using a Pierce bicinchoninic acid (BCA) protein assay kit (Thermo Fisher Scientific). Protein was precipitated in 90% methanol and pelleted (5,000 × *g*, 15 min). The supernatants were discarded, and the protein pellets were suspended in 8 M urea with 100 mM Tris (pH = 8), 10 mM Tris(2-carboxyethylphosphine) (TCEP), and 40 mM chloroacetamide (CAA). Lysates were diluted to 1.5 M urea with 50 mM Tris (pH = 8). Trypsin (Promega) was added (1:50 [enzyme/protein]), and the reaction mixture was incubated overnight at ambient temperature. Ratios were based on protein concentrations (8 h, 0.5 mg; 12 h, 0.7 mg; 16 h, 1.0 mg; 20 h, 1 mg; 24 h, 1 mg; 28 h, 1.5 mg; 32 h, 2 mg). An additional aliquot of trypsin (1:100 [enzyme/protein]) was added in the morning for an hour. Peptides were isolated and desalted by the use of a Strata-X column (Phenomenex) (polymeric reversed phase; 10 mg/ml). Desalting columns were equilibrated with 1 ml of 100% acetonitrile (ACN) followed by 1 ml of 0.2% formic acid. Samples were acidified with trifluoroacetic acid (TFA) and loaded onto the equilibrated Strata-X columns, which were then washed with 1 ml 0.2% formic acid. Peptides were eluted into clean tubes with 650 μl 40% ACN followed by 550 μl 70% ACN, dried, and reconstituted in 0.2% formic acid. Peptide concentrations were measured prior to MS analysis using the Pierce quantitative colorimetric peptide assay (Thermo).

### LC-MS/MS proteomic analysis.

For each time point, 1.5 μg/μl of peptide solution was analyzed using a nano-LC system coupled to an Orbitrap Fusion Lumos mass spectrometer (Thermo Scientific, San Jose, CA). Injections were loaded onto a reversed-phase nano-ultra-high-performance LC column for chromatographic separation prior to MS analysis. Columns were prepared in-house from a bare fused-silica capillary column (75-μm inner diameter, 360-μm outer diameter) with a laser-pulled electrospray tip. The tips were etched with 48% hydrofluoric acid, and the column was packed with bridged ethylene hybrid (BEH) C_18_ particles (Waters, Milford, MA) (130-Å pore size, 1.7-μm particle size) using an ultra-high-pressure column packing station at maximum pressure (between 20,000 and 30,000 lb/in^2^) (136). Columns were installed on a Dionex Ultimate 3000 nano-UHPLC system (Thermo Fisher, Sunnyvale, CA) and equilibrated with mobile phase A (0.2% formic acid–water) and mobile phase B (0.2% formic acid–70% ACN). Peptides were separated over 90 min using the following gradient: 0 min, 0% solvent B; 7 min, 8% solvent B; 75 min, 60% solvent B; 77 to 81 min, 100% solvent B; 82 to 90 min, 0% solvent B (including the time needed for column reequilibration). Eluting peptides were subjected to MS analysis following positive-mode electrospray ionization. Global MS settings utilized advanced precursor determination to enable nearly complete utilization of the rapid acquisition rates of the Orbitrap (137). MS survey scans were performed using the Orbitrap (240 K resolution, AGC target value of 1e−6, and 50 ms maximum injection time). Monoisotopic precursor selection and dynamic exclusion (15 s) were enabled. The MS/MS analysis was performed by the use of a 0.7 *m/z* isolation with the quadrupole, normalized higher-energy collisional dissociation (HCD) data at 25%, and analysis of fragment ions in the ion trap using the “Turbo” scan speed (200 to 1,200 *m/z* scan range, AGC target set at 3e−4, and 11-ms maximum injection time).

### Analysis of proteome data.

Thermo RAW files were processed by the use of MaxQuant (version 1.6.0.16) (138). Searches were performed against a target decoy database of Bacillus subtilis proteins, including isoforms (UniProt; downloaded 23 August 2017). The precursor search tolerance was set at 4.5 ppm, and the product mass tolerance was set to 0.4 Da. Quantitation was performed as label-free quantitation (LFQ) with an LFQ minimum ratio of 1. A match between runs was used to quantify peptides with a label minimum ratio count of 1. Search parameters included fixed modification for carbamidomethylation of cysteine residues, variable modification for oxidation of methionine, *N*-terminal acetylation, and a maximum of 2 missed cleavages. The peptide spectral match (PSM) false-discovery rate (FDR) and protein FDR were both set to 1%. Normalization is embedded in the MaxQuant LFQ logarithm (139).

### Statistical analyses.

All -omics data were subjected to one-way ANOVA to corroborate the significance of the temporal changes. Principal-component analyses for both transcriptome and proteome data sets were done using dudi.pca in the ade4 package (R statistics) (140). Correlations between proteome data and transcriptome data were calculated using cor.test (R statistics) to display Pearson's product moment correlation coefficients.

### Detection of biofilm subpopulations using fluorescent reporters.

B. subtilis reporter strains *amyE*::P*alsS-gfp ΔcomI* and *amyE*::P*acoA-gfp ΔcomI* were constructed as previously described (141, 142) using the following primers to amplify the promoter regions of *alsSD* and *acoABCL* operons: FalsS (GACGGTCTCATGTCAGGAGGCTGCGTCATGTTC [5′ → 3′]), RalsS (GTCGGTCTCTCGAACACCCTCACTCCTTATTATGCATTTTAAA), FacoA (GACGGTCTCATGTCAAAGATTTCCAAGGAAATAAATACGTC), and RacoA (GTCGGTCTCTCGAAGTACCTTGGTTATTTGCCCCG). B. subtilis NCIB3610 (WT), *amyE*::P*alsS-gfp ΔcomI*, and *amyE*::P*acoA-gfp ΔcomI* were grown in MSgg medium for up to 32 h at 37°C. Planktonic cultures (OD_600_ of ∼0.06) and biofilms were subjected to mild sonication and dilution. Cells were spotted onto thin agarose pads (1% agarose–MSgg medum) on multiwell slides (MP Biomedicals, LLC), covered with a cover slip, and imaged on an Olympus IX83 inverted microscope using a 60× oil immersion objective. Images were captured using a Hamamatsu digital CCD camera with bright-field (BF) and green fluorescent protein (GFP) cube filters and were analyzed using MetaMorph (Molecular Devices, Inc.).

### Measurement of protein content in biofilms.

Levels of protein in biofilm pellets, as well as in the spent media of biofilms, were measured using the Bradford assay. In brief, biofilms were subjected to centrifugation followed by flash-freezing and stored at −80°C prior to analysis. The frozen pellicles were thawed on ice. Lysis buffer (1.5 ml) was added to each sample in a 15-ml conical vial for pellet resuspension. The pellet resuspension was subjected to sonication (QSonica model Q125; Qsonica, Newtown, CT) (30% amplitude; 125 watts; 20 kHz) in 3 pulses of 10 s each with 25 s of resting on ice between pulses. A 20-μl volume of the resultant lysate was transferred to a new 1.5-ml microcentrifuge tube and mixed with 80 μl of 0.15 M sodium chloride. A 1-ml volume of Bradford solution was added to the tube, and the reaction mixture was subjected to vortex mixing. The reaction mixture was incubated for 2 min at room temperature. The optical density at 595 nm (OD_595_) of the reaction mixture was measured using a spectrophotometer (Genesys 20; Thermo Scientific). Biofilm supernatant (i.e., spent medium) was obtained by centrifugation and was subjected directly to sodium chloride treatment followed by Bradford solution treatment. A standard curve was created using 0.5 mg/ml bovine serum albumin (BSA).

### Measurement of extracellular DNA (eDNA) concentrations.

Extracellular DNA (eDNA) associated with biofilms was quantified using PicoGreen spectrophotometric assay. In brief, pellicles (biofilms) were transferred to a small petri dish using custom-made stainless-steel mesh and immediately resuspended in 1 ml of spent medium from the same well. The pellicle was broken down by pipetting followed by two passages through a syringe needle (23 gauge; 0.6 mm by 25 mm). The resuspension was transferred to a 1.5-ml microcentrifuge tube. A 1-ml volume of of the remaining spent medium in the well was transferred to another 1.5-ml microcentrifuge tube. The two tubes were subjected to centrifugation for 5 min at maximum speed (16,100 × *g*) using a tabletop centrifuge. The supernatant from each tube was filtered through a 0.2-μm-pore-size nylon filter (Millipore) and stored at −20°C until analysis. The spectrophotometric assay (Quant-iT PicoGreen dsDNA reagent and kits) was performed per the instructions of the manufacturer (Invitrogen). Lambda DNA standard provided in the kit was used for eDNA quantification.

### Measurement of iron concentrations in spent biofilm medium.

Spent medium of biofilm was subjected to total iron measurement using an iron assay kit (Sigma-Aldrich catalog no. MAK025) per the manufacturer’s instructions.
