# Supplementary figures and images for: Metabolic Remodeling during Biofilm Development of Bacillus subtilis
Source: mBio. 2019 May 21;10(3):e00623-19. doi: 10.1128/mBio.00623-19 (PMC6529636; doi:10.1128/mBio.00623-19)

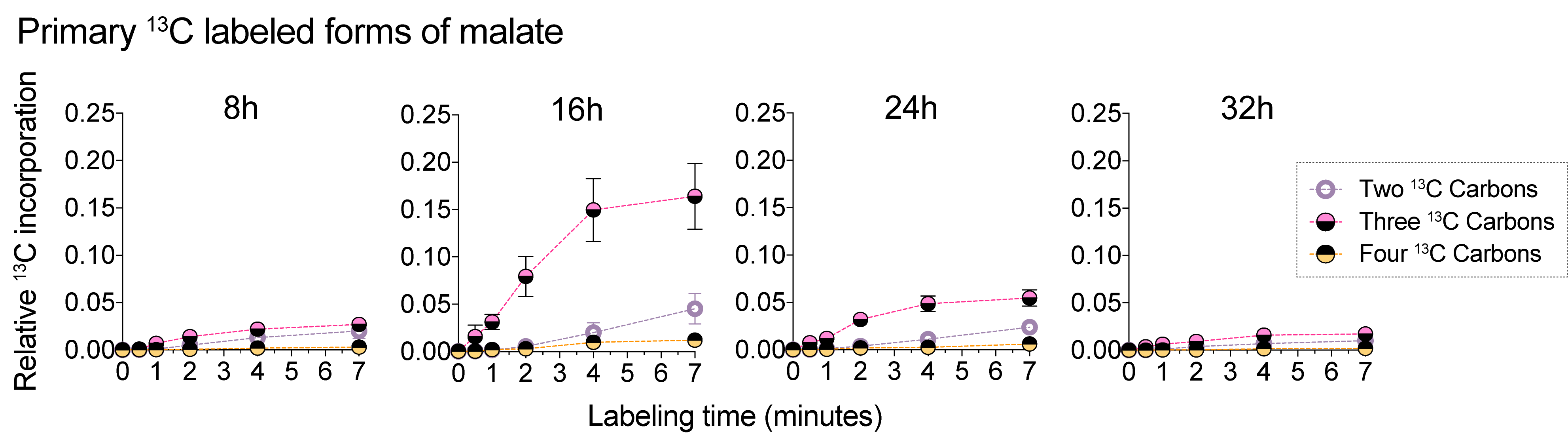

Supplement: FIG S1 [file mBio.00623-19-sf001.tif]

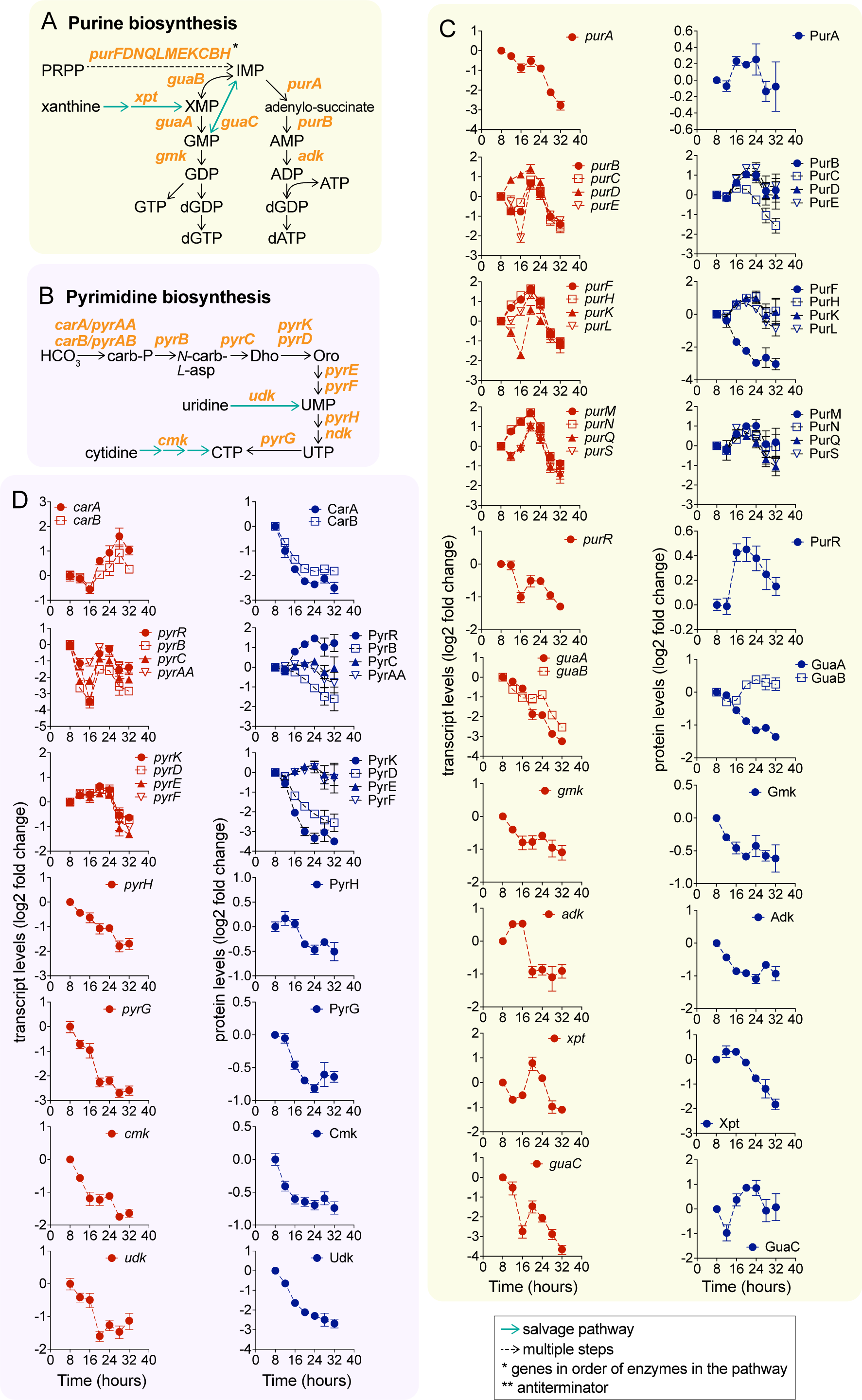

Supplement: FIG S2 [file mBio.00623-19-sf002.tif]

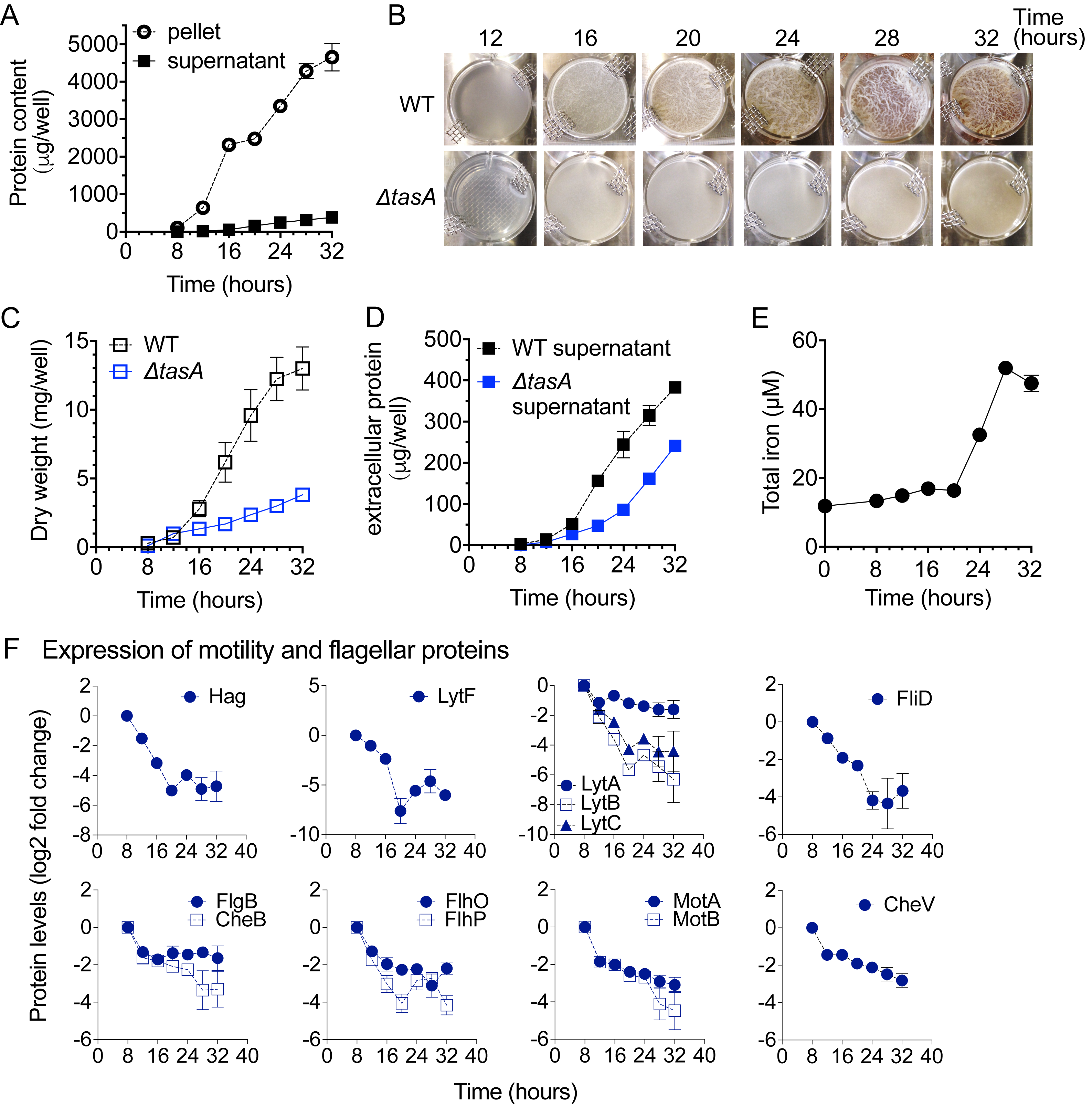

Supplement: FIG S3 [file mBio.00623-19-sf003.tif]

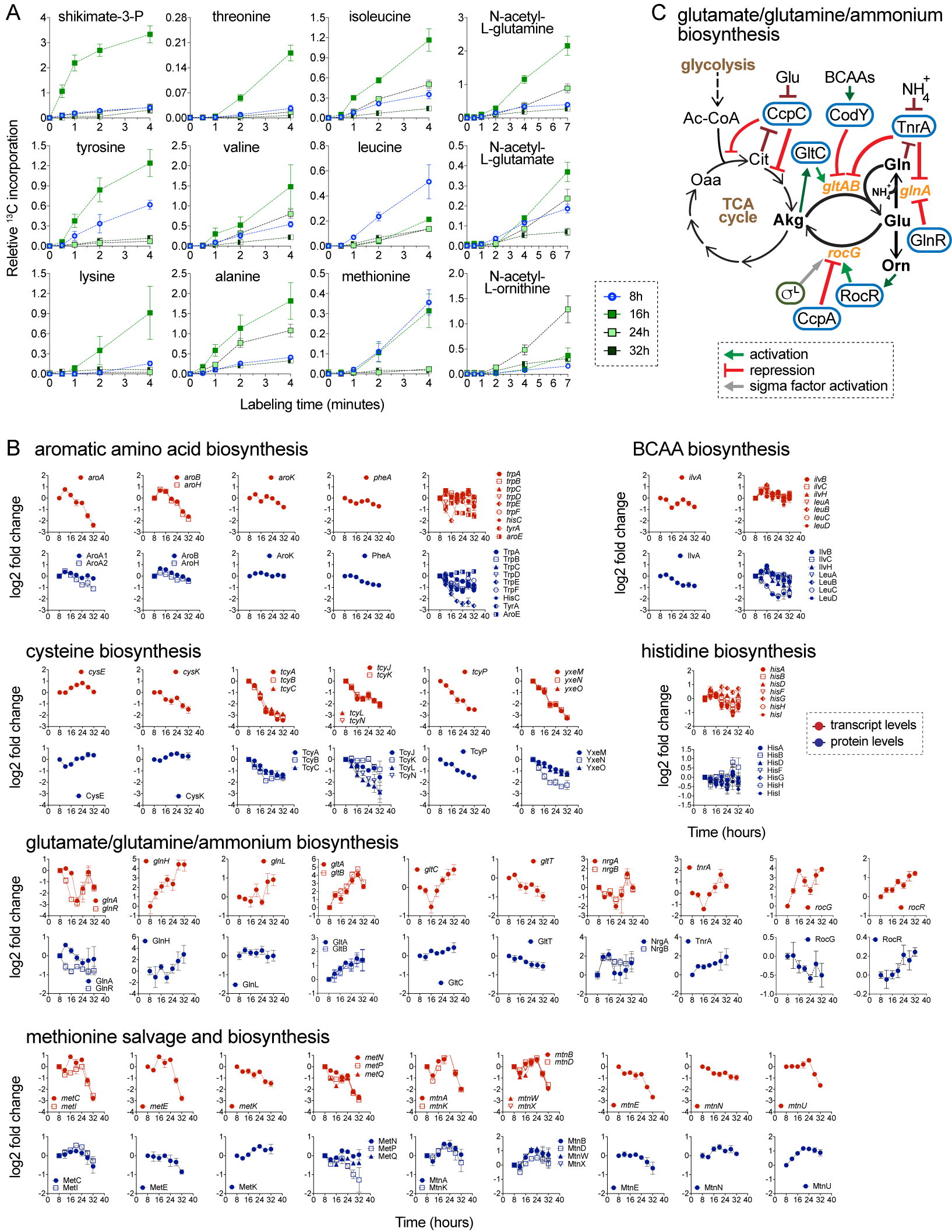

Supplement: FIG S4 [file mBio.00623-19-sf004.tif]

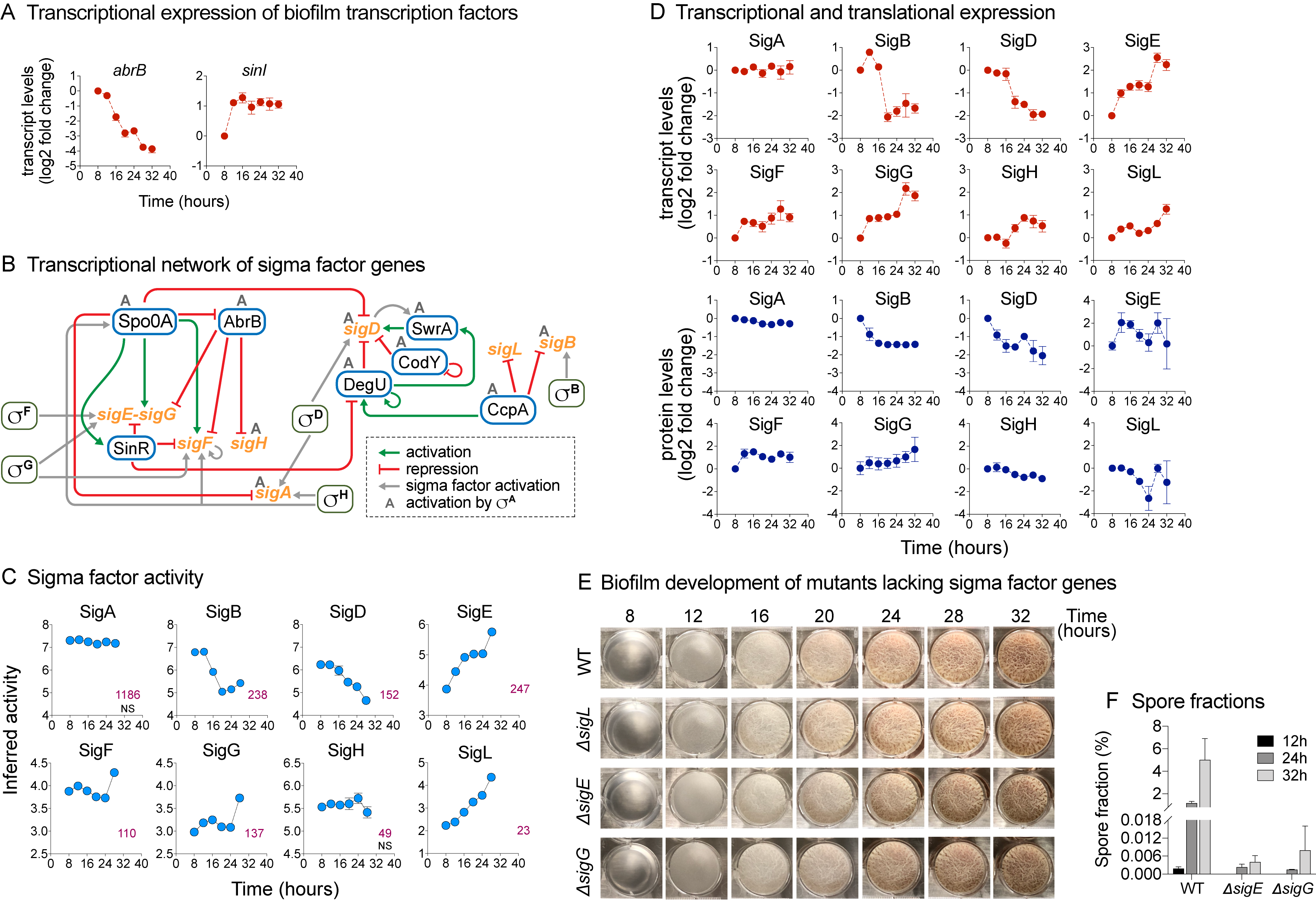

Supplement: FIG S5 [file mBio.00623-19-sf005.tif]

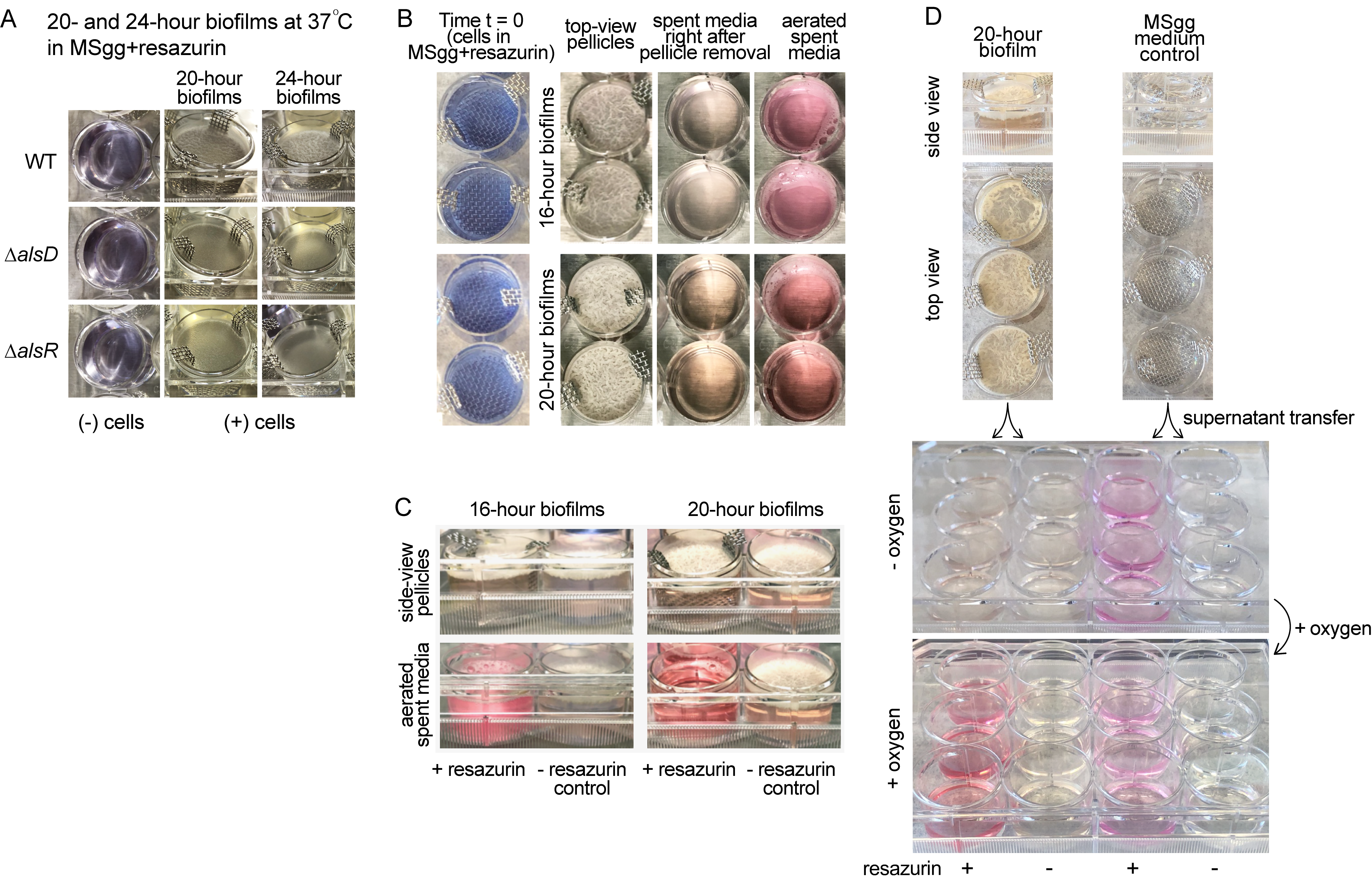

Supplement: FIG S7 [file mBio.00623-19-sf007.tif]

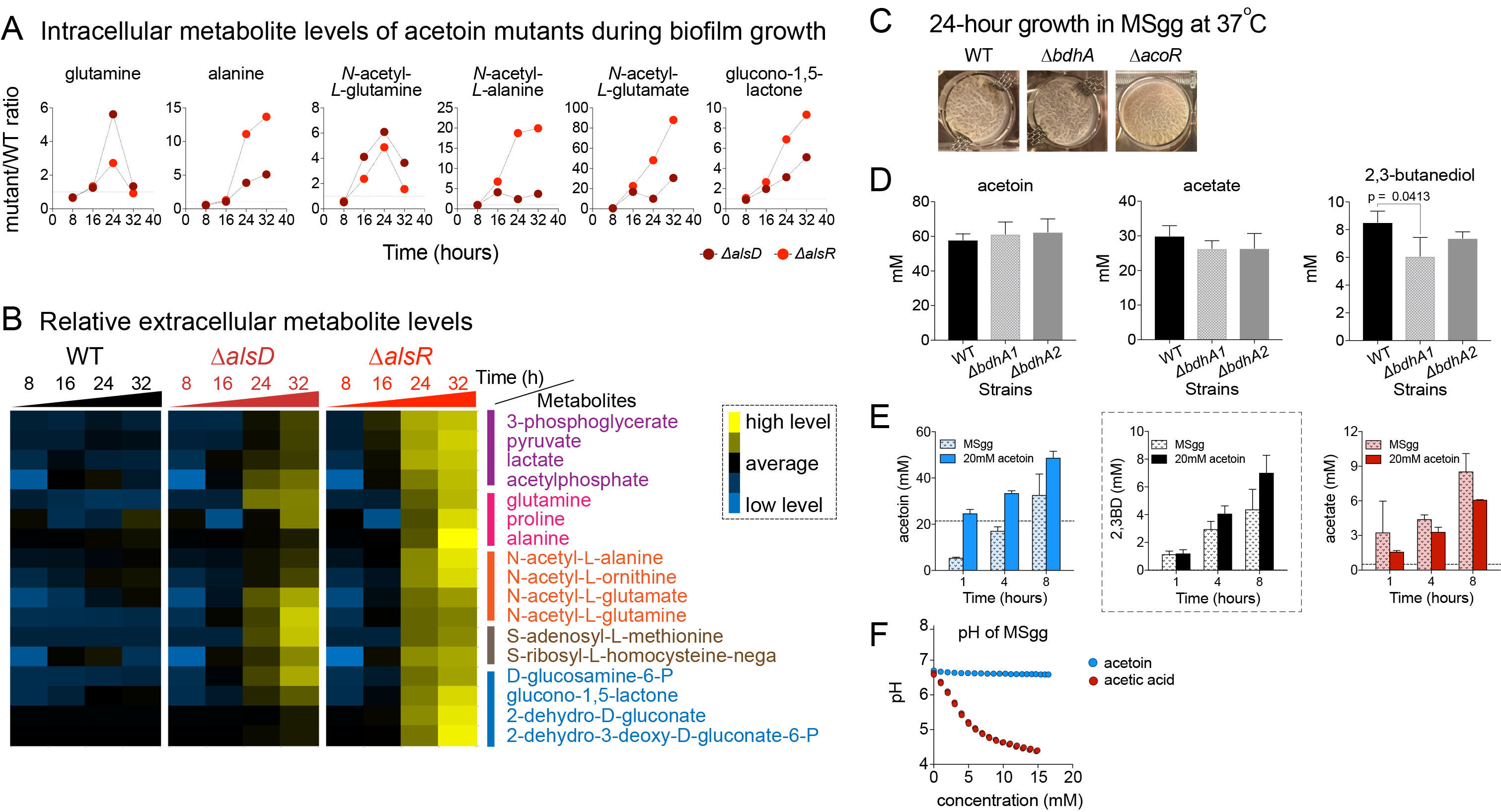

Supplement: FIG S9 [file mBio.00623-19-sf009.tif]

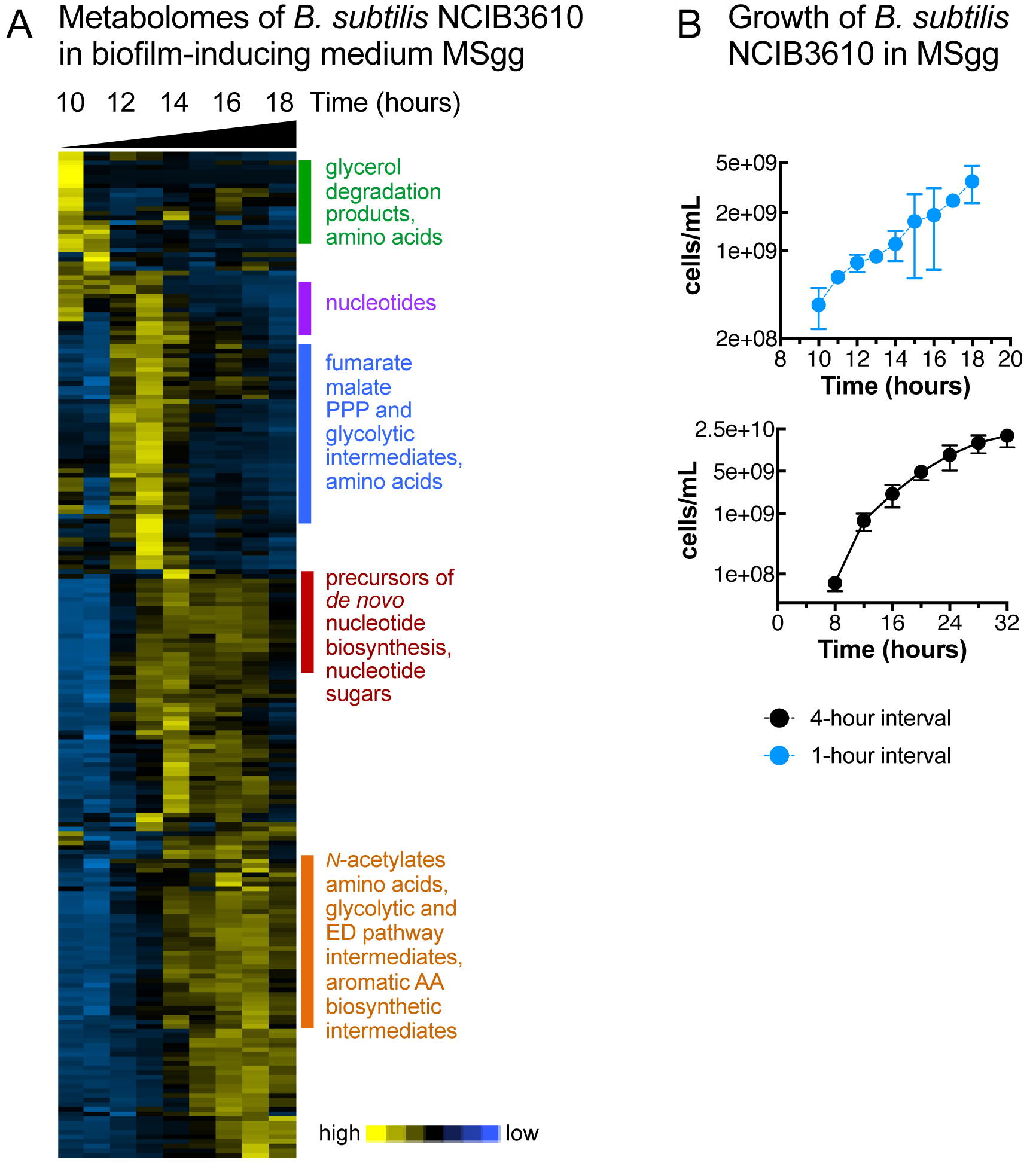

Supplement: FIG S10 [file mBio.00623-19-sf010.tif]
